# DHCR24 Drives Ovarian Cancer Chemoresistance Through Lipid Raft-mediated P-gp Stabilization and STAT3 Activation

**DOI:** 10.7150/ijbs.128173

**Published:** 2026-03-17

**Authors:** Xin Fu, Zhaosong Wang, Zhining Yang, Yue Xu, Qiuping Dong, Yanfen Cui, Ke Wang

**Affiliations:** 1 Tianjin Medical University Cancer Institute and Hospital, National Clinical Research Center for Cancer, Tianjin, 300060, China.; 2 Key Laboratory of Cancer Prevention and Therapy, Tianjin, 300060, China.; 3 Tianjin's Clinical Research Center for Cancer, Tianjin, 300060, China.; 4 Department of Gynecologic Oncology, Tianjin Medical University Cancer Institute and Hospital, Tianjin, 300060, China.; 5 Laboratory Animal Center, Tianjin Medical University Cancer Institute and Hospital, Tianjin, 300060, China.; 6 Laboratory of Tumor Cell Biology, Tianjin Medical University Cancer Institute and Hospital, Tianjin, 300060, China.; 7 Public Laboratory, Tianjin Medical University Cancer Institute and Hospital, Tianjin, 300060, China.

**Keywords:** DHCR24, cholesterol, STAT3, P-gp, ovarian cancer, chemotherapy resistance

## Abstract

**Objective:**

To investigate the role and mechanism of DHCR24 in chemoresistance of ovarian cancer and to identify potential therapeutic targets for overcoming treatment resistance.

**Methods:**

We integrated bioinformatic analysis of GEO datasets and clinical survival data from KMplot to identify chemoresistance-associated genes. DHCR24 expression and function were systematically evaluated using cisplatin-resistant cell lines (A2780/DDP, SKOV3/DDP), patient-derived primary cells, xenograft models, and clinical specimens through molecular biology techniques, immunohistochemistry, and functional assays. Mechanistic studies employed RNA interference, cholesterol modulation, lipid raft disruption with MβCD, cycloheximide chase assays, and STAT3 pathway inhibition.

**Results:**

DHCR24 was consistently upregulated in chemoresistant ovarian cancer models and significantly correlated with poor patient survival. Genetic or pharmacological inhibition of DHCR24 restored chemosensitivity *in vitro* and *in vivo*, while its overexpression induced cross-resistance to multiple chemotherapeutic agents. Mechanistically, DHCR24 enhanced cholesterol biosynthesis, which stabilized lipid raft microdomains to promote P-gp protein stability and facilitate STAT3 membrane recruitment and activation. Furthermore, activated STAT3 transcriptionally upregulated DHCR24 expression, establishing a positive feedback loop that perpetuates the chemoresistant phenotype.

**Conclusion:**

DHCR24 drives chemoresistance through a cholesterol-dependent circuit that stabilizes drug efflux pumps and activates pro-survival signaling, identifying DHCR24 as a promising therapeutic target for overcoming chemotherapy resistance in ovarian cancer.

## Introduction

Ovarian cancer maintains its position as the most lethal gynecologic malignancy worldwide, presenting clinicians with significant therapeutic challenges. Current statistics show that more than 70% of patients are diagnosed at advanced stages (FIGO stage III or IV), when the disease has already metastasized throughout the peritoneal cavity 1. Even with aggressive treatment combining cytoreductive surgery and platinum-based chemotherapy, the majority of these patients experience disease recurrence within just 18 months of initial therapy, highlighting the critical need for more effective treatment strategies. This disheartening clinical outcome mainly stems from the rapid development of chemotherapy resistance, a complex and multi-faceted phenomenon that poses the greatest obstacle to improving patients' survival 2.

The mechanism of chemotherapy resistance in ovarian cancer involves complex and coordinated changes at multiple biological levels. At the genetic level, tumor cells accumulate mutations and gain a survival advantage under therapeutic pressure. Epigenetic reprogramming alters gene expression patterns without changing DNA sequences, thereby achieving adaptive responses to chemotherapy 3-5. Perhaps most interestingly, metabolic reprogramming allows cancer cells to rewire their energy production and biosynthetic pathways to defend against cytotoxic damage 6-8. Among these metabolic adaptations, cholesterol metabolism disorders have recently become a hallmark of treatment-resistant cancers in various tumor types, including breast cancer, prostate cancer and pancreatic cancer 9-11. Cholesterol, traditionally viewed as a structural component of cell membranes, is now recognized as a key regulator of cancer cell signaling, membrane fluidity, and drug efflux 12, 13. In ovarian cancer specifically, altered cholesterol homeostasis has been implicated in platinum resistance, but the specific enzymatic drivers of this process remain poorly characterized. This knowledge gap is particularly significant because cholesterol metabolic enzymes represent potentially druggable targets. The identification and characterization of these enzymatic regulators could provide crucial insights into chemoresistance mechanisms while revealing novel therapeutic vulnerabilities that might be exploited to improve treatment outcomes for ovarian cancer patients facing limited options after developing platinum resistance.

DHCR24 (24-dehydrocholesterol reductase), the key terminal enzyme in the Bloch cholesterol biosynthesis pathway, catalyzes the reduction of desmosterol to cholesterol 14. Beyond its canonical metabolic function in cholesterol production, emerging evidence reveals that DHCR24 exhibits fascinating roles in cellular processes including oxidative stress response, endoplasmic reticulum homeostasis, and even modulation of inflammatory signaling pathways 15-17. In the context of cancer biology, elevated DHCR24 expression has been correlated with poor prognosis in several malignancies, including breast and prostate cancers 18-24, where it has been suggested to promote therapeutic resistance through mechanisms that remain largely undefined. However, the functional significance of DHCR24 in ovarian cancer pathogenesis and chemoresistance remains virtually unexplored, representing a significant knowledge gap in the field. The constitutive activation of STAT3 has emerged as a consistent hallmark of chemoresistant ovarian cancer, strongly correlating with poor patient outcomes and treatment failure. As a key transcriptional regulator, activated STAT3 drives the expression of anti-apoptotic proteins (such as BCL-2 and survivin), effectively creating a multi-faceted defense against chemotherapy-induced cell death 25. While the downstream consequences of STAT3 activation are well-characterized, the upstream mechanisms sustaining its activity in recurrent ovarian cancer remain incompletely understood.

To address these questions, we employed an integrated research strategy combining bioinformatic discovery, functional validation, and clinical correlation. Through an analysis of GEO datasets and KMplot survival validation, we identified DHCR24 as a top candidate gene associated with platinum resistance, a finding corroborated in cisplatin-resistant cell lines, patient-derived cells, and chemoresistant tumor tissues. Genetic knockdown of DHCR24 restored chemosensitivity *in vivo*, reducing xenograft tumor volumes, while its overexpression induced cross-resistance to multiple agents. Mechanistically, DHCR24-driven cholesterol synthesis stabilized P-gp in lipid rafts while simultaneously promoting STAT3 membrane recruitment and nuclear translocation. Crucially, we discovered that activated STAT3 transcriptionally upregulates DHCR24, thereby completing a self-reinforcing circuit that further amplifies cholesterol synthesis.

## Materials and Methods

### Bioinformatic analysis

Differentially expressed genes (DEGs) related to ovarian cancer chemoresistance were identified from three GEO databases (GSE15709, GSE1926 and GSE3001). KMplot (https://kmplot.com/analysis/), which includes an ovarian cancer cohort of 1,287 patients with RNA-seq and clinical data, was used to study the relationships between DEGs expression levels and overall survival (OS) and progression-free survival (PFS) prognosis of patients with ovarian cancer, and optimal expression cutoff was determined automatically using maximally selected rank statistics. Kaplan-Meier curves generated using ggplot2-based engine. In addition, raw RNA-seq data from TCGA dataset and GEO datasets of GSE14407, GSE18520 and GSE36668 were used to analyze the expression levels of DHCR24 mRNA in ovarian tissues and ovarian cancer tissues. The Human Protein Atlas (HPA, https://www.proteinatlas.org/) was used to analyze the expression levels of DHCR24 protein in ovarian tissues and ovarian cancer tissues.

### Patients and tissue samples

In addition to the public databases, 128 cases of epithelial ovarian cancer (EOC) tissues were obtained from the Cancer Institute of Tianjin Medical University and the Department of Pathology of its affiliated hospitals. All tissues were examined by pathologists in accordance with the standards of the World Health Organization, and none of the patients had received any other treatments before the surgery. The staging and grading of cancer were determined by the International Federation of Obstetrics and Gynecology (FIGO) criteria. The use of these specimens and patient information has been approved by the Cancer Institute of Tianjin Medical University and the Hospital Ethics Committee. Among 128 EOC patients, 103 underwent platinum-based chemotherapy (paclitaxel/cisplatin or paclitaxel/carboplatin). Based on therapeutic response criteria, these patients were stratified as chemosensitive group (n = 64, no recurrence beyond 6 months post-treatment or sustained remission) and chemoresistant group (n = 39, disease progression during therapy or recurrence within 6 months post-chemotherapy).

### Mice xenograft model

Female Balb/c-nu/nu mice (5-6 weeks) from Beijing HFK Bioscience Co. were housed under specific pathogen-free conditions at the Laboratory Animal Center of Tianjin Medical University Cancer Institute and Hospital. To establish subcutaneous tumor models, 5×10⁶ SKOV3/DDP cells transfected with either shctrl or shDHCR24 were inoculated into the right dorsal flank of the mice, and these mice were randomized into two groups (n = 5 per group) approximately 10 days post-inoculation when tumors reached about 5 mm in diameter. The groups received daily intraperitoneal injections of either normal saline or cisplatin (2 mg/kg). In the second experiment, mice bearing wild-type SKOV3/DDP tumors were randomized into four groups (n = 5 per group) at the same tumor size benchmark. The groups received daily intraperitoneal injections of: 1) normal saline, 2) cisplatin (2 mg/kg), 3) Triparanol (30 mg/kg), or 4) cisplatin (2 mg/kg) plus Triparanol (30 mg/kg). In the third experiment, mice bearing wild-type SKOV3/DDP tumors were randomized into four groups (n=5 per group) at the same tumor size benchmark. The groups received daily intraperitoneal injections of: 1) normal saline, 2) cisplatin (2 mg/kg), 3) Stattic (5 mg/kg), or 4) cisplatin (2 mg/kg) plus Stattic (5 mg/kg). Tumor growth was monitored regularly throughout the study. All mice were euthanized at the experimental endpoint of 3 weeks, upon which tumors were excised, and their volume and weight were measured. Tumor volume was calculated using the formula: (length × width²)/2. All animal procedures were approved by Tianjin Cancer Hospital and conducted in accordance with the NIH Guide for the Care and Use of Laboratory Animals.

### Immunohistochemistry staining

Immunohistochemistry staining was performed as described previously 26. Tissue sections underwent sequential dewaxing in xylene followed by rehydration through graded ethanol solutions. Antigen retrieval was performed using high-pressure heat-mediated epitope unmasking in 10 mM citrate buffer (pH 6.0). Endogenous peroxidase activity was quenched with 3% hydrogen peroxide. Specimens were then probed overnight at 4°C with primary antibodies against DHCR24 (1:100, Cat#sc-398938, Santa), p-STAT3 (1:200, Cat#9145, CST), Ki-67 (1:100, Cat#sc-23900, Santa) or Cleaved-caspase 3 (1:400, Cat#9661, CST) followed by 1-hour incubation with species-matched secondary antibodies at 37°C. Immunocomplexes were visualized via 5-minute DAB chromogenic development. DHCR24 expression quantification employed a semiquantitative histoscore system: positive cell percentage (1: 0-25%, 2: 26-50%, 3: 51-75%, 4: 76-100%) multiplied by staining intensity (0: negative, 1: weak, 2: moderate, 3: strong). Final scores were used to dichotomize samples into high-expression (≥ 6) and low-expression (< 6) cohorts. For expression quantification of p-STAT3, Ki-67 and Cleaved-caspase 3, expression levels were quantified based on the percentage of immunopositive cells.

### Cell culture and treatment

Human EOC cell lines SKOV3 and A2780 were obtained from ATCC (American Type Culture Collection), while their drug-resistant counterparts, SKOV3/DDP and A2780/DDP, were acquired from Wuhan Procell Life Science and Technology Co., Ltd. (Wuhan, China). All cells were maintained in RPMI 1640 medium supplemented with 10% fetal bovine serum, 100 U/ml penicillin, and 100 μg/ml streptomycin, and cultured at 37°C in a humidified 5% CO₂ incubator. To preserve drug resistance, SKOV3/DDP and A2780/DDP cells were continuously exposed to cisplatin (DDP) at 1 μg/mL and 0.6 μg/mL, respectively. Prior to experiments, the medium was replaced with DDP-free culture medium three days in advance. All cell lines were authenticated by short tandem repeat profiling and confirmed free of Mycoplasma contamination.

### Ascites tumor cell isolation

Ascitic fluid (approximately 100 mL) was collected from ovarian cancer patients with confirmed ascites via ultrasound-guided paracentesis. The sample was immediately transported to the laboratory and pre-filtered through a 70 μm cell strainer. After filtration, the fluid was centrifuged at 1000 r/min for 10 minutes to pellet the cellular components. The resulting cell precipitate was gently resuspended in 1 mL of pre-cooled red blood cell lysis buffer and incubated at room temperature for 10 minutes. This was followed by the addition of 10 mL of pre-cooled, calcium- and magnesium-free PBS and another centrifugation step under the same conditions. The pelleted cells were then washed twice with calcium- and magnesium-free PBS and once with complete RPMI 1640 medium, which was supplemented with 10% fetal bovine serum (Hyclone, USA), 100 U/mL penicillin, and 100 μg/mL streptomycin (Invitrogen, USA). Finally, the cells were adjusted to an appropriate concentration and seeded into 96-well plates for subsequent cisplatin treatment.

### Plasmid construction and cell infection

To downregulate DHCR24, specific shRNA targeting gene was cloned into the pLKO.1 plasmid. The sequence used was CCGCGTGTGAAACACTTTGAA. For DHCR24 overexpression, its full-length coding sequence was amplified by PCR using the primers 5'-GAGAGAATTCGCCACCATGGAGCCCGCCGTGT-3' (forward) and 5'-GATCGACTCGAGTCAGTGCCTGGCGGCCTT-3' (reverse), and subsequently inserted into the pCDH-CMV-MCS-Puro lentiviral vector. All constructed plasmids were verified by DNA sequencing. Lentiviruses were produced in HEK293T cells by co-transfecting the respective plasmid with the psPAX2 and pMD2.G packaging plasmids using polyethyleneimine. The viral supernatant was harvested 48 hours post-transfection and used to infect the target cancer cells. Knockdown and overexpression efficiencies were confirmed by real-time PCR and western blot.

### Quantitative Real-Time PCR

Total RNA was extracted with Trizol and reverse-transcribed into cDNA using HiScript II Q RT SuperMix (Vazyme, China). Quantitative PCR was subsequently performed with AceQ qPCR SYBR Green Master Mix (Vazyme, China) on a real-time PCR system. The following primer sequences were shown in Supplementary Table 1. Gene expression levels were quantified using the 2^(-ΔΔCt) method.

### Western blot analysis

Cells were lysed in ice-cold SDS lysis buffer, and protein concentrations were determined using the BCA assay. Equal amounts of proteins were separated by 10% SDS-PAGE and subsequently transferred to polyvinylidene fluoride (PVDF) membranes via a semi-dry system. After blocking with 5% non-fat milk in TBST, the membranes were incubated overnight at 4 °C with the following primary antibodies: DHCR24 (1:1000, Cat#sc-398938, Santa), p-STAT3 (1:1000, Cat#9145, CST), STAT3(1:1000, Cat#9132, CST), P-gp (1:1000, Cat#sc-55510, Santa Cruz), and GAPDH (1:5,000, Cat#2118, CST ). This was followed by incubation with a secondary antibody for 1 h at 37 °C. Protein bands were visualized using an enhanced chemiluminescence (ECL) reagent (Millipore, USA), and their grayscale values were quantified with ImageJ software, using GAPDH as the loading control.

### CCK-8 assay for cell viability and proliferation

Cell proliferation was assessed using a Cell Counting Kit-8 (CCK-8, Bimake, USA) according to the manufacturer's instructions. Briefly, cells were seeded into 96-well plates at a density of 1,000 cells per well. After the incubation period, 10 µL of CCK-8 solution was added to each well, followed by further incubation for 3 hours at 37 °C. The absorbance was then measured at 450 nm to determine cell viability.

### Colony Formation Assay

For the colony formation assay, cells were seeded in 12-well plates at a density of 1,000 cells per well. Following the incubation, the resulting colonies were washed with PBS, fixed with methanol, and stained with crystal violet. The number of colonies was then counted under an inverted microscope, and representative images were captured.

### Immunofluorescence Staining

Cells grown on coverslips were fixed with 4% paraformaldehyde for 15 minutes at room temperature and subsequently permeabilized with 0.1% Triton X-100 for 10 minutes. After blocking with 5% bovine serum albumin (BSA) for 1 hour, the cells were incubated overnight at 4°C with the designated primary antibody diluted in blocking buffer: P-gp (1:100), STAT3 (1:100) and p-STAT3 (1:100). Following washes, the samples were incubated with a fluorophore-conjugated secondary antibody for 1 hour at room temperature in the dark. Nuclei were counterstained with DAPI (4',6-diamidino-2-phenylindole), and the coverslips were mounted onto glass slides using an anti-fade mounting medium. Images were finally captured and analyzed using a fluorescence microscope.

### Cholesterol detection

The concentration of free cholesterol in cells was measured using a cholesterol detection kit in accordance with the manufacturer's instructions (ab65390, Abcam). Cell lysates were prepared and subsequently incubated with cholesterol oxidase. The resulting fluorescence intensity was recorded with excitation and emission wavelengths set at 538 and 587 nm, respectively. The test for cholesterol esters initially converted them into free cholesterol through cholesterol esterase. Total cholesterol represents the sum of free and esterified cholesterol.

### Rhodamine 123 efflux assay

Cells in the logarithmic growth phase were collected and resuspended at a density of 1×10⁶ cells/mL in culture medium containing 1.0 μg/mL Rhodamine 123. The cells were incubated at 37°C with 5% CO₂ for 30 minutes to allow dye uptake. After centrifugation and three washes with PBS, the cells were re-incubated in dye-free medium at 37°C. Samples were then collected, washed twice with PBS, resuspended in 200 μL of PBS, and immediately analyzed by flow cytometry. The efflux function of P-gp was evaluated based on the percentage of Rhodamine 123-positive cells and the mean fluorescence intensity.

### ChIP Assay Procedure

Chromatin immunoprecipitation was performed using the BeyoChIP™ Enzymatic ChIP Assay Kit. SKOV3/DDP cells were cross-linked with 1% formaldehyde for 10 min at 37 °C, followed by glycine quenching. Cells were washed with cold PBS containing protease inhibitors, scraped, and aliquoted (~4×10⁶ cells per tube). After centrifugation, pellets were resuspended in Buffer A and then Buffer B. Chromatin was fragmented using MNase (37 °C, 20 min) and sonicated (3 cycles of 20 sec) after resuspension in ChIP Buffer. The lysate was cleared by centrifugation and diluted to 0.5 mL with ChIP Buffer. A 10 μL aliquot was saved as Input. The remainder was pre-cleared with Protein A/G Magnetic Beads for 30 min at 4 °C, then incubated with primary antibody overnight at 4 °C. Immune complexes were captured with beads for 1 h and washed sequentially with low-salt, high-salt, LiCl, and TE buffers. Complexes were eluted twice with 150 μL Elution Buffer. Cross-links were reversed by adding NaCl and heating at 65 °C for 2 h. Samples were then treated with EDTA, Tris, and proteinase K at 45 °C for 1 h. Purified DNA was used for subsequent analysis.

### Luciferase Activity Analysis

A dual-luciferase reporter assay was performed to evaluate STAT3 activity. Briefly, cells were plated in 96-well plates at a density of 2×10³ cells per well and co-transfected with a STAT3-responsive firefly luciferase plasmid and a constitutive Renilla luciferase control plasmid (both provided by Shanghai Genechem). After 24 hours, the cells were transfected with DHCR24 overexpression plasmid or vehicle control for a further 24 hours. Following treatment, the cells were lysed, and luminescence was detected using the Promega Dual-Luciferase® Reporter Assay System. The firefly luminescence readings were normalized to the Renilla values to account for transfection efficiency.

### Statistical methods

All statistical analyses were performed using SPSS 19.0. The expression levels of DHCR24 across different EOC groups were compared using a standard two-tailed t-test, while the association between DHCR24 expression and patient survival was evaluated by Kaplan-Meier analysis with the log-rank test. A p-value of less than 0.05 was considered statistically significant.

## Results

### DHCR24 expression level is associated with chemoresistance in ovarian cancer cells

**Figure 1:**
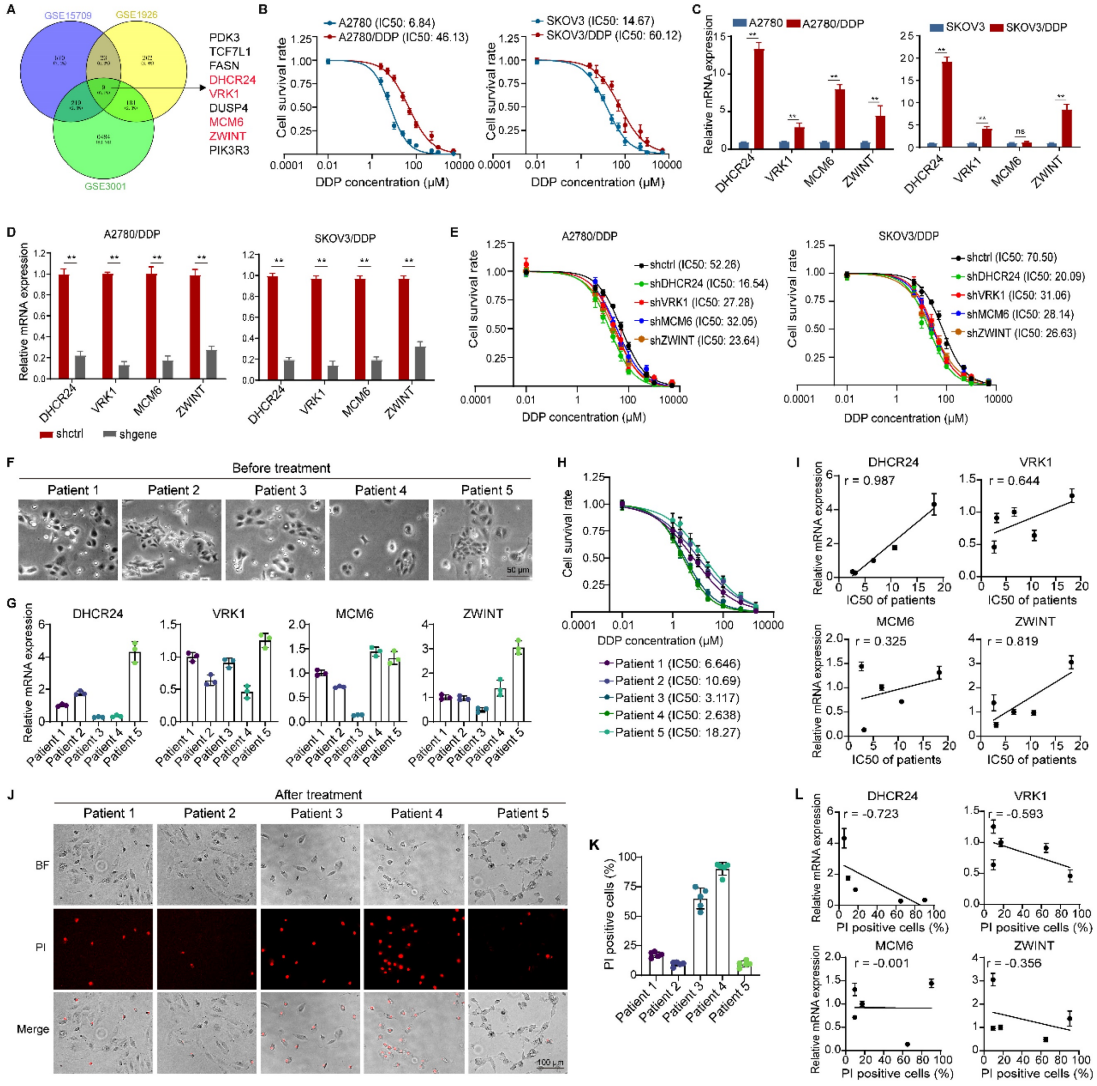
** DHCR24 expression level is associated with chemoresistance in ovarian cancer cells. (A)** Differentially expressed genes (DEGs) related to ovarian cancer chemoresistance were identified from three GEO databases (GSE15709, GSE1926 and GSE3001). **(B)** IC50 values of DDP in ovarian cancer cells measured by CCK8 assay at 48 h, validating drug-resistant cell models. **(C)** mRNA expression levels of DHCR24, VRK1, MCM6, and ZWINT in drug-resistant and parental ovarian cancer cells were detected by qRT-PCR. **(D)** Knockdown efficiency of DHCR24, VRK1, MCM6 and ZWINT in drug-resistant ovarian cancer cells. **(E)** IC50 values of DDP at 48 h in ovarian cancer drug-resistant cells after knockdown of DHCR24, VRK1, MCM6 or ZWINT. **(F)** Morphological images of primary tumor cells from five ovarian cancer patients. **(G)** Expression levels of DHCR24, VRK1, MCM6 and ZWINT in primary tumor cells from F. **(H)** IC50 values of DDP in primary tumor cells from F at 48 h. **(I)** Correlations between IC50 values of DDP and expression levels of DHCR24, VRK1, MCM6 and ZWINT in primary tumor cells. **(J)** Propidium iodide (PI) staining of dead primary tumor cells after 48 h treatment with 5 μM DDP. **(K)** Percentage of PI-positive cells in each primary tumor cell line. **(L)** Correlation between PI-positive cell proportion and expression of DHCR24, VRK1, MCM6, and ZWINT. All data are shown as the mean ± SD; ns, non-significant, ** p < 0.01, n = 3.

To investigate the mechanisms underlying chemoresistance in ovarian cancer, we analyzed differentially expressed genes (DEGs) from three GEO databases associated with ovarian cancer resistance. We identified 9 DEGs consistently detected across all datasets (Fig. 1A). Subsequently, we utilized the KMplot database to analyze the relationship between the expression levels of these 9 genes and the survival prognosis of ovarian cancer patients. The results revealed that four of these genes (DHCR24, VRK1, MCM6, ZWINT) were significantly associated with both overall survival (Fig. S1A) and progression-free survival (Fig. S1B) in patients. Therefore, we hypothesized that these four genes might be more strongly correlated with chemoresistance in ovarian cancer. We further examined the expression levels of these four genes in cisplatin-resistant ovarian cancer cell lines (A2780/DDP and SKOV3/DDP, Fig. 1B) and their corresponding parental cells. The results demonstrated that the expression levels of all four genes were upregulated in the resistant cells, with DHCR24 exhibiting the most significant upregulation (Fig. 1C and Fig. S2A). Downregulation of each gene (Fig. 1D and Fig. S2B) increased the sensitivity of the ovarian cancer cells to cisplatin, and the effect of DHCR24 knockdown was the most pronounced (Fig. 1E). Furthermore, we isolated primary cancer cells from 5 human ovarian cancer tissue samples (Fig. 1F) and measured the expression levels of the four genes (Fig. 1G) and the IC50 value for DDP (Fig. 1H) in these primary cells. We observed a positive correlation between the expression level of each gene and the IC50 value for DDP across these cells, with the strongest correlation observed for DHCR24 (Fig. 1I). Concurrently, propidium iodide (PI) staining (Fig. 1J) was used to assess the cell death rate following DDP treatment in each primary cell line (Fig. 1K), which also demonstrated that the expression level of DHCR24 exhibited the most significant correlation with the cell death rate (Fig. 1L). Therefore, these collective results indicate that the expression level of DHCR24 is associated with chemoresistance in ovarian cancer cells.

### Altering DHCR24 expression affects chemosensitivity in ovarian cancer cells

**Figure 2:**
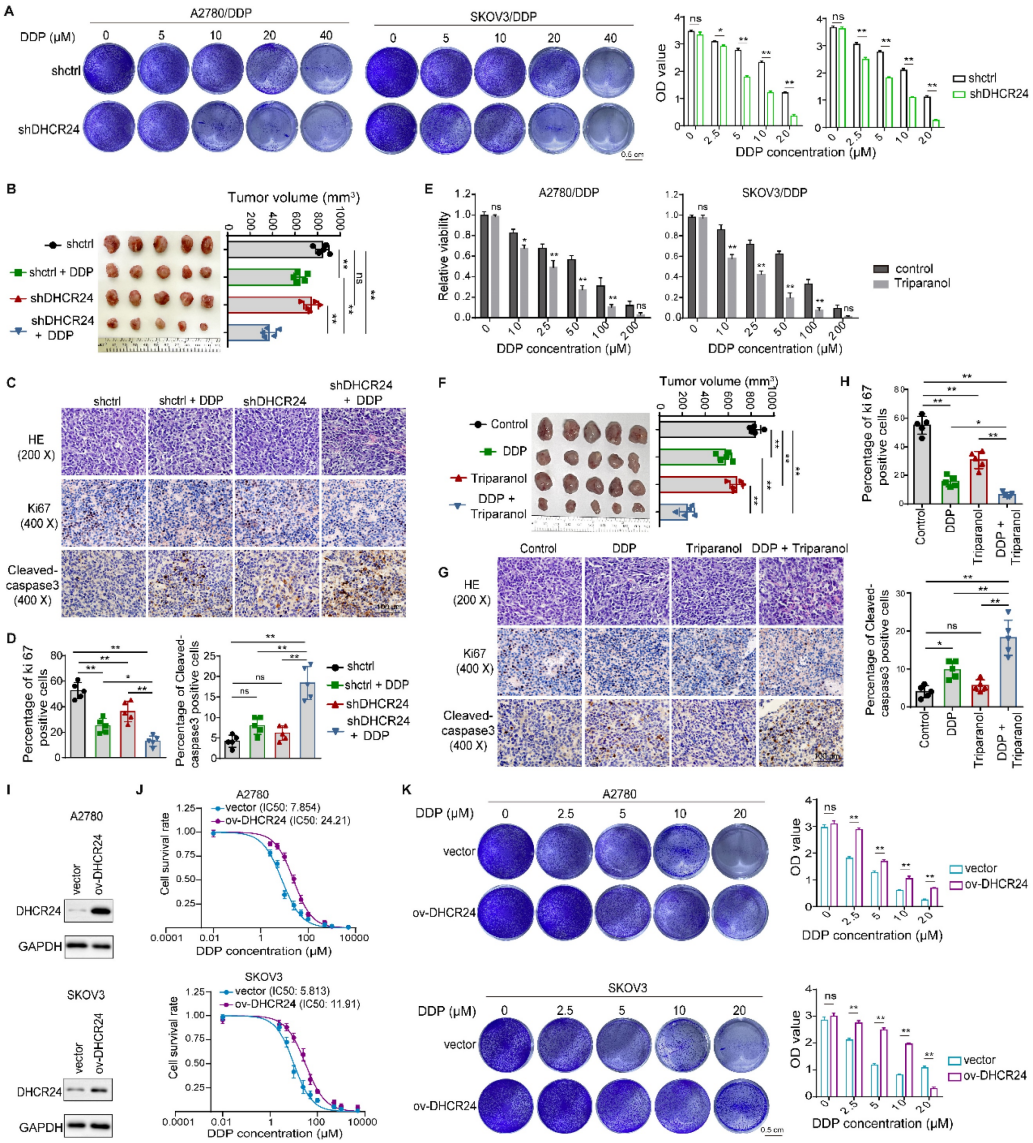
** Modulation of DHCR24 expression affects chemosensitivity in ovarian cancer cells. (A)** Effect of DHCR24 knockdown on DDP sensitivity in drug-resistant ovarian cancer cells was measured by cell proliferation assay. **(B)** Impact of DHCR24 knockdown on DDP sensitivity was detected in A2780/DDP xenograft mice. **(C)** Representative H&E and IHC staining of tumors from (B). **(D)** Statistical results of Ki67 and cleaved-caspase3 in tumor tissues from (C). **(E)** Effect of DHCR24 inhibition with Triparanol on DDP sensitivity was detected by CCK8. **(F)** Effect of DHCR24 inhibition with Triparanol on DDP sensitivity was detected in A2780/DDP xenograft mice. **(G)** Representative H&E and IHC staining of tumors from (F). **(H)** Statistical results of Ki67 and cleaved-caspase3 in tumor tissues from (G). **(I)** Western blot validation of DHCR24 overexpression in ovarian cancer cells. **(J)** IC50 values of DDP at 48 h in ovarian cancer cells with DHCR24 overexpression. **(K)** Effect of DHCR24 overexpression on DDP sensitivity in ovarian cancer cells was detected by cell proliferation assay. All data are shown as the mean ± SD; ns, non-significant, * p < 0.05, ** p < 0.01; *in vitro*: n = 3, *in vivo*: n = 5.

To further elucidate the role of DHCR24 in ovarian cancer drug sensitivity, we confirmed through *in vitro* cell proliferation assays that DDP significantly enhanced the inhibition of cell proliferation in DHCR24-knockdown cells (Fig. 2A). Furthermore, using a xenograft mouse model established with A2780/DDP cells, we demonstrated that DHCR24 knockdown significantly increased tumor sensitivity to DDP. This was evidenced by the smallest tumor volume in the DHCR24-knockdown group treated with DDP (Fig. 2B), accompanied by a significant decrease in the Ki67 proliferation index and a significant increase in cleaved-caspase 3-positive cells within the tumors (Fig. 2C-D). Additionally, application of the DHCR24 inhibitor Triparanol similarly increased tumor cell sensitivity to DDP (Fig. 2E). Likewise, using the A2780/DDP xenograft model, we found that inhibiting DHCR24 significantly enhanced tumor sensitivity to DDP. The group treated with a combination of DDP and Triparanol exhibited the smallest tumor volume (Fig. 2F), along with a significantly reduced Ki67 proliferation index and a significantly increased number of cleaved-caspase 3-positive cells in the tumors (Fig. 2G-H). Moreover, Triparanol treatment did not cause significant body weight loss or obvious pathological lesions in vital organs, and serum liver/kidney function markers remained within normal ranges (Fig. S2C-E). Conversely, overexpression of DHCR24 in ovarian cancer cells (Fig. 2I, Fig. S3A) markedly suppressed their sensitivity to DDP (Fig. 2J and Fig. S3B). Consistent with this, cell proliferation assays also showed that DDP-induced inhibition of cell proliferation was significantly attenuated in DHCR24-overexpressing cells (Fig. 2K and Fig. S3C). Moreover, DHCR24 overexpression not only suppressed sensitivity to DDP but also reduced sensitivity to other chemotherapeutic agents, including paclitaxel (Fig. S3D) and epirubicin (Fig. S3E). In conclusion, these results collectively demonstrate that DHCR24 expression levels regulate the sensitivity of ovarian cancer cells to chemotherapeutic drugs.

### Expression of DHCR24 in ovarian cancer tissues and its relationship with patient chemotherapy response

**Figure 3:**
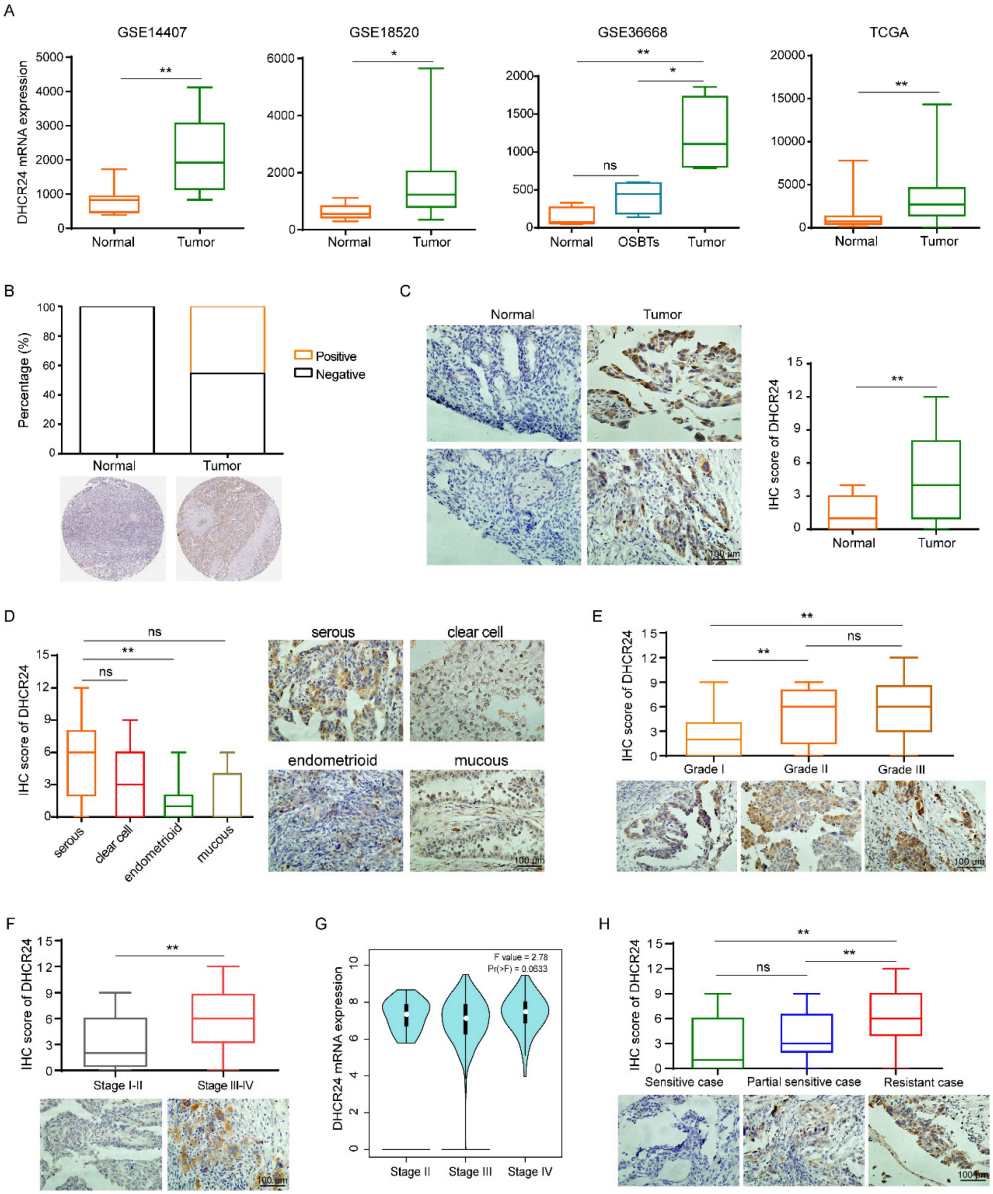
** Expression of DHCR24 in ovarian cancer tissues and its relationship with patient chemotherapy response. (A)** DHCR24 mRNA expression in ovarian cancer versus normal tissues in public GEO and TCGA databases. **(B)** DHCR24 protein expression in ovarian cancer versus normal tissues in HPA. **(C)** IHC staining of DHCR24 protein in ovarian cancer and normal tissues. **(D)** DHCR24 protein levels across ovarian cancer histological subtypes. **(E)** DHCR24 protein expression stratified by pathological stage. **(F)** DHCR24 protein expression stratified by clinical stage. **(G)** DHCR24 mRNA expression stratified by clinical stage. **(H)** Association between DHCR24 protein expression and chemotherapy response in ovarian cancer patients. All data are shown as the mean ± SD; ns, non-significant, * p < 0.05, ** p < 0.01.

Given our previous findings that DHCR24 expression is associated with chemoresistance in ovarian cancer, we specifically sought to understand the expression profile of DHCR24 in ovarian cancer tissues and its relationship with chemotherapy response in patients. To better understand the role of DHCR24 in ovarian cancer, we first analyzed data from public GSE and TCGA databases. This analysis revealed that DHCR24 expression was significantly upregulated in ovarian cancer tissues compared with normal ovarian tissues (Fig. 3A-B). Concurrently, we performed IHC staining on tumor tissues from 128 ovarian cancer patients. This also confirmed the upregulation of DHCR24 in ovarian cancer tissues (Fig. 3C). Among the histological subtypes, DHCR24 expression was highest in serous ovarian carcinoma and lowest in endometrioid ovarian carcinoma (Fig. 3D). Furthermore, the expression level of DHCR24 in ovarian cancer tissues was closely correlated with tumor pathological grade (Fig. 3E) and clinical stage (Fig. 3F). Higher DHCR24 protein expression was observed in tumors with poorer differentiation (higher grade) and more advanced clinical stage. TCGA data analysis also demonstrated higher DHCR24 mRNA expression levels in tumors with more advanced clinical stage (Fig. 3G). Importantly, the protein expression level of DHCR24 in ovarian cancer tissues was associated with patient chemotherapy response. High DHCR24 protein expression was detected in chemoresistant tumor tissues (Fig. 3H). The above results indicate that the expression level of DHCR24 in ovarian cancer tissues is related to tumor progression and response to chemotherapy.

### DHCR24 regulates chemosensitivity in ovarian cancer cells through cholesterol synthesis-dependent lipid raft stabilization

**Figure 4:**
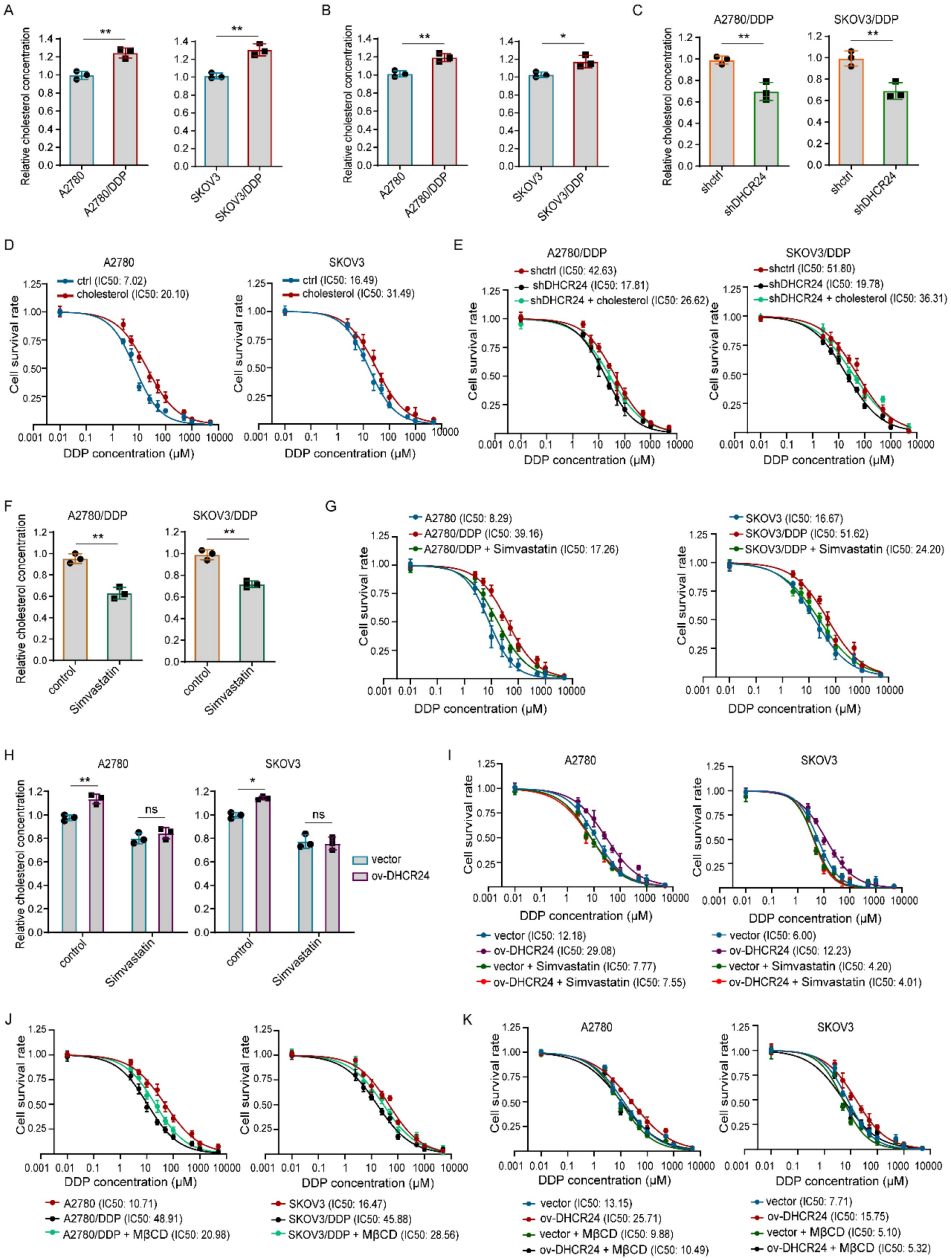
** DHCR24 modulates chemosensitivity in ovarian cancer by regulating cholesterol biosynthesis. (A)** Intracellular cholesterol levels in drug-resistant and parental ovarian cancer cells. **(B)** Extracellular cholesterol levels in conditioned medium from drug-resistant and parental cells. **(C)** Effect of DHCR24 knockdown on intracellular cholesterol in drug-resistant cells. **(D)** Impact of cholesterol (10 μM) on DDP sensitivity. **(E)** Exogenous cholesterol reversed chemosensitivity induced by DHCR24 knockdown in drug-resistant cells. **(F)** Inhibition of cholesterol synthesis by Simvastatin (10 μM) in drug-resistant cells. **(G)** Effect of Simvastatin on the DDP sensitivity of drug-resistant cells. **(H)** Simvastatin (10 μM) suppressed cholesterol elevation in DHCR24-overexpressing ovarian cancer cells. **(I)** Effect of Simvastatin (10 μM) on the DDP sensitivity of ovarian cancer cells overexpressing DHCR24. **(J)** Effect of MβCD (2.5 mM) on the DDP sensitivity of drug-resistant cells. **(K)** Effect of MβCD (2.5 mM) on the DDP sensitivity of ovarian cancer cells overexpressing DHCR24. All data are shown as the mean ± SD; ns, non-significant, * p < 0.05, ** p < 0.01, n = 3.

Having established that DHCR24 expression influences the chemosensitivity of ovarian cancer cells, we next investigated the underlying mechanism. Given that DHCR24 primarily functions as a reductase involved in the cholesterol biosynthesis pathway, we first examined whether DHCR24 could affect tumor cell chemosensitivity by increasing cholesterol levels in ovarian cancer resistant cell lines. Indeed, we detected significantly higher cholesterol levels both intracellularly and in the culture medium of resistant cells compared with their parental counterparts (Fig. 4A-B). Knockdown of DHCR24 significantly reduced intracellular cholesterol content in resistant cells (Fig. 4C). Furthermore, supplementation with exogenous cholesterol inhibited the sensitivity of ovarian cancer cells to DDP (Fig. 4D) and prevented the increase in chemosensitivity induced by DHCR24 silencing in resistant cells (Fig. 4E). Conversely, inhibiting cholesterol synthesis in resistant cells using Simvastatin (Fig. 4F) increased their sensitivity to DDP (Fig. 4G). Importantly, in the presence of statins, DHCR24 overexpression was unable to increase cholesterol levels or confer chemoresistance (Fig. 4H and I). Given that cholesterol is a critical component of lipid rafts and that drug resistance-associated proteins such as P-gp are predominantly localized in these microdomains, we disrupted lipid raft integrity by depleting cholesterol with methyl-β-cyclodextrin (MβCD). The results showed that treatment of MβCD enhanced the sensitivity of SKOV3/DDP and A2780/DDP cells to DDP (Fig. 4J), and effectively reversed the chemoresistance induced by DHCR24 overexpression (Fig. 4K). Collectively, these results demonstrate that DHCR24 regulates chemosensitivity in ovarian cancer cells through cholesterol synthesis-dependent lipid raft stabilization.

### DHCR24 regulates ovarian cancer chemosensitivity through a mechanism mediated by lipid raft-dependent stabilization of P-gp

**Figure 5:**
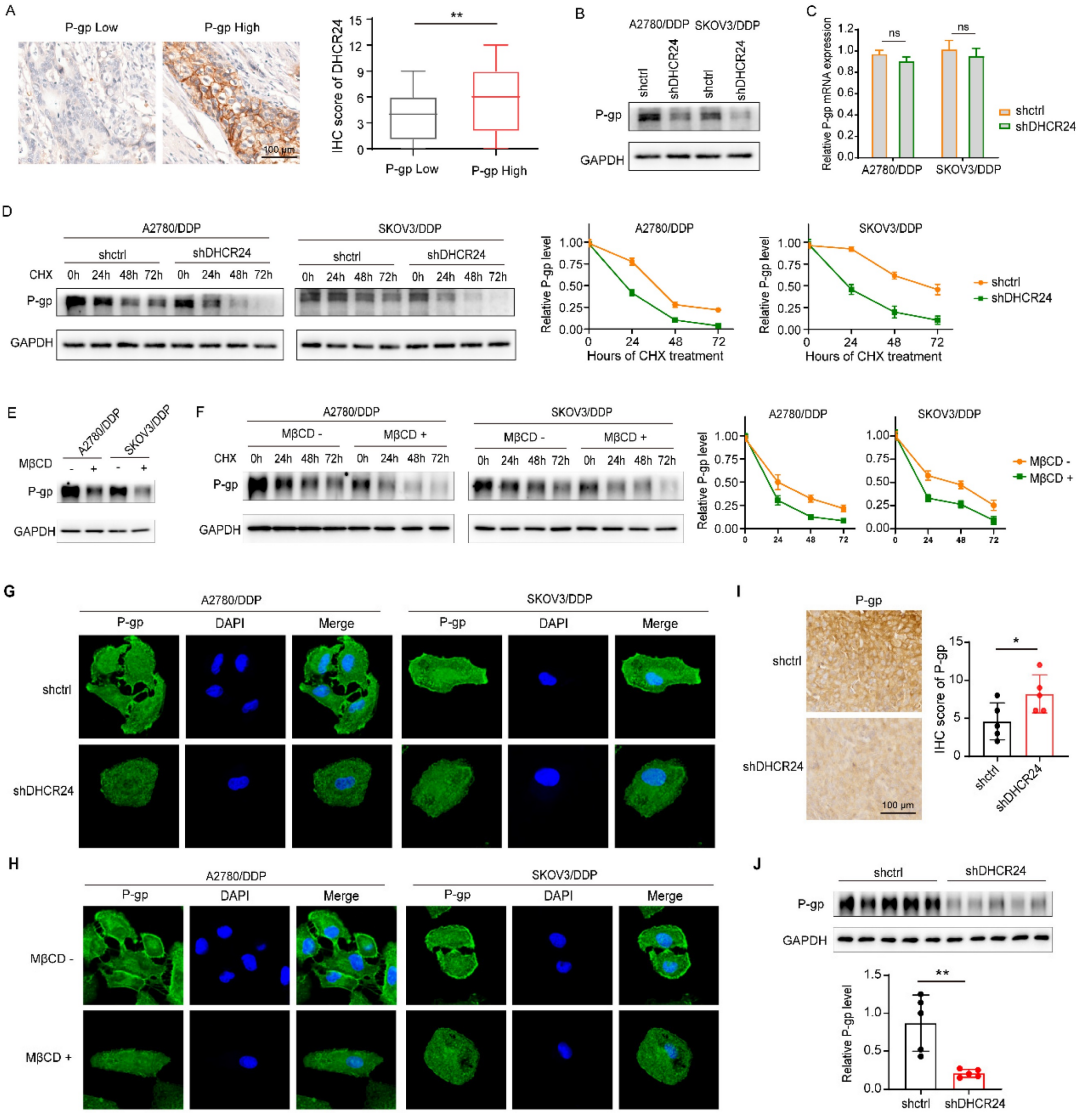
** DHCR24 regulates P-gp protein stability via cholesterol-rich lipid rafts in ovarian cancer cells. (A)** Immunohistochemical staining of ovarian cancer tissues showing co-elevated expression of DHCR24 and P-gp. **(B)** Western blot analysis of P-gp protein expression in A2780/DDP and SKOV3/DDP cells after DHCR24 knockdown. **(C)** Quantitative PCR analysis of P-gp mRNA levels in DHCR24-silenced A2780/DDP and SKOV3/DDP cells. **(D)** Cycloheximide (CHX, 50 **μ**g) chase assay evaluating P-gp protein stability upon DHCR24 knockdown in A2780/DDP and SKOV3/DDP cells. **(E)** Western blot analysis of P-gp expression following lipid raft disruption via MβCD-mediated cholesterol depletion. **(F)** CHX chase assay assessing P-gp protein stability after MβCD treatment. **(G)** Immunofluorescence staining of P-gp (green) and nuclei (DAPI, blue) in A2780/DDP cells after DHCR24 silencing. **(H)** Immunofluorescence staining of P-gp (green) and nuclei (DAPI, blue) in A2780/DDP cells after MβCD treatment. **(I)** Immunohistochemical staining of P-gp in xenograft tumor tissues from the shDHCR24 and shctrl groups (same cohort as Fig. 2B). **(J)** Western blot analysis of P-gp protein levels in xenograft tumor tissues from the shDHCR24 and shctrl groups.

Given that cholesterol serves as a critical structural component of lipid rafts and that drug resistance-associated proteins such as P-gp are predominantly localized within these microdomains 27, we sought to determine whether DHCR24-induced chemoresistance in ovarian cancer involves P-gp regulation. Immunohistochemical analysis of ovarian cancer tissues revealed co-elevated expression of DHCR24 and P-gp (Fig. 5A). Consistent with this, silencing DHCR24 in resistant cell lines significantly suppressed P-gp protein expression (Fig. 5B), without altering its mRNA levels (Fig. 5C). Cycloheximide (CHX) chase assays further demonstrated that DHCR24 knockdown markedly accelerated P-gp degradation (Fig. 5D). To assess the functional role of lipid rafts, we disrupted membrane integrity through cholesterol depletion using MβCD. This intervention not only reduced P-gp protein expression (Fig. 5E) but also impaired its stability (Fig. 5F). Cellular immunofluorescence staining confirmed that both DHCR24 silencing (Fig. 5G) and MβCD treatment (Fig. 5H) diminished membrane localization of P-gp. Furthermore, using the P-gp substrate Rhodamine 123 and flow cytometry, we assessed P-gp efflux activity. We observed that either DHCR24 silencing or MβCD treatment elevated intracellular Rhodamine 123 accumulation, whereas cholesterol supplementation attenuated this effect (Fig. S4). Validation in mouse xenograft models (from Fig. 2B) via IHC (Fig. 5I) and western bloting (Fig. 5J) consistently showed that DHCR24 knockdown suppressed P-gp expression *in vivo*. Taken together, these results demonstrate that DHCR24 enhances P-gp protein stability by modulating cholesterol-rich lipid raft microdomains.

### DHCR24 modulates ovarian cancer cell sensitivity to chemotherapy by regulating STAT3 signaling pathway activation

**Figure 6:**
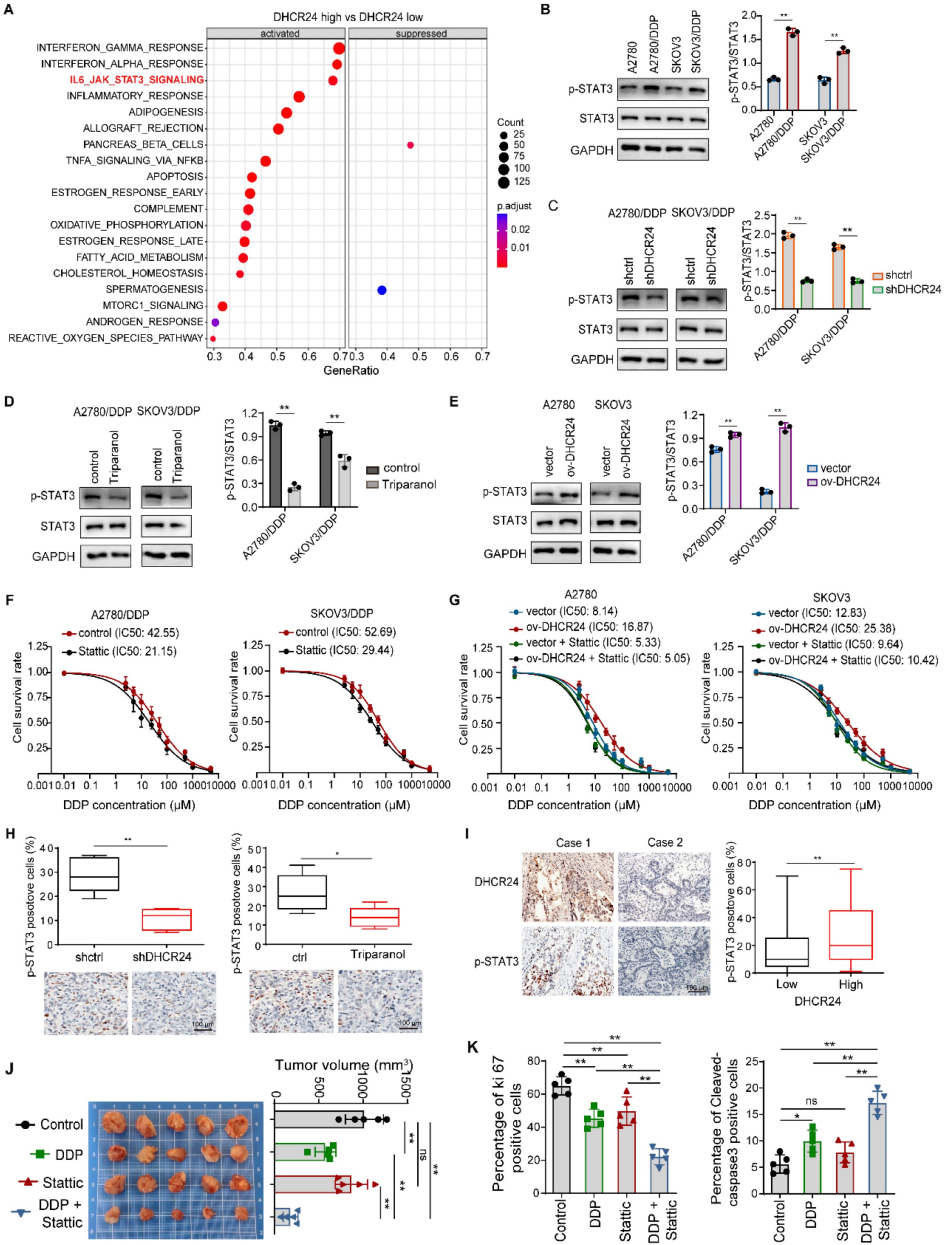
** DHCR24 modulates chemosensitivity through STAT3 signaling in ovarian cancer. (A)** Enrichment analysis of ovarian cancer pathways stratified by DHCR24 mRNA expression using TCGA database. **(B)** STAT3 activation (phosphorylation) in drug-resistant and parental cells detected by Western blot. **(C)** Effect of DHCR24 knockdown on STAT3 activation in drug-resistant cells. **(D)** Triparanol-mediated DHCR24 inhibition suppresses STAT3 activation. **(E)** Effect of DHCR24 overexpression on STAT3 activation in parental cells. **(F)** Effect of Stattic on the DDP sensitivity of drug-resistant cells. **(G)** Effect of Stattic on the DDP sensitivity of ovarian cancer cells overexpressing DHCR24. **(H)** Immunohistochemistry was used to detect the effect of inhibiting DHCR24 on STAT3 signal activation in mouse transplanted tumor tissues. **(I)** Immunohistochemistry was used to detect the correlation between the expression level of DHCR24 protein and the level of p-STAT3 in human ovarian cancer tissues. **(J)** Effect of STAT3 inhibition with Stattic on DDP sensitivity was detected in A2780/DDP xenograft mice. **(K)** Statistical results of Ki67 and cleaved-caspase3 in tumor tissues from (J). All data are shown as the mean ± SD; * p < 0.05, ** p < 0.01, n = 3.

To further elucidate the mechanism of DHCR24, we performed enrichment analysis using ovarian cancer data from TCGA based on DHCR24 mRNA expression levels, and found that among canonical signaling pathways, DHCR24 mRNA expression was most significantly associated with STAT3 pathway activation (Fig. 6A). Our Western blot results confirmed that chemoresistant cells exhibited elevated STAT3 activation compared with parental cells (Fig. 6B). Importantly, DHCR24 knockdown in resistant cells suppressed STAT3 activation (Fig. 6C), recapitulated by DHCR24 Triparanol (Fig. 6D). Conversely, DHCR24 overexpression upregulated STAT3 activation (Fig. 6E), collectively indicating that DHCR24 promotes STAT3 pathway activation. Simultaneously, STAT3-regulated genes (Survivin, MMP9, CyclinD1, BCL-2) were upregulated in resistant cells (Fig. S5A), a phenomenon reversed by DHCR24 silencing (Fig. S5B). As STAT3 activation regulates chemosensitivity, STAT3 inhibition (Stattic) indeed reversed chemoresistance in our model (Fig. 6F). We therefore investigated if DHCR24 influences chemosensitivity via STAT3. In DHCR24-overexpressing cells, pharmacological inhibition of STAT3 activation abrogated the effect; compared with control cells, DHCR24 overexpression no longer affected tumor cell sensitivity to chemotherapy drugs when STAT3 was inhibited (Fig. 6G). This suggests DHCR24 specifically affects chemosensitivity through STAT3 regulation. To further validate the regulatory role of DHCR24 on STAT3 activation *in vivo*, we utilized a mouse xenograft model. Both knockdown of DHCR24 expression and inhibition of its activity suppressed STAT3 activation in the tumors (Fig. 6H). Furthermore, in human ovarian cancer tissues, DHCR24 protein levels positively correlated with p-STAT3 levels (Fig. 6I), and the same result was obtained in primary cells (Fig. S5C). Additionally, Stattic significantly increased the sensitivity of tumor cells to DDP *in vivo*. Mice treated with a combination of DDP and Stattic exhibited the smallest tumor volume (Fig. 6J), along with a significantly reduced Ki67 proliferation index and a significantly increased number of cleaved-caspase 3-positive cells in the tumors (Fig. 6K). Together, these results demonstrate that DHCR24 modulates tumor cell sensitivity to chemotherapeutic agents by regulating STAT3 signaling pathway activation.

### DHCR24 governs STAT3 activation by regulating lipid raft stability via cholesterol synthesis

**Figure 7:**
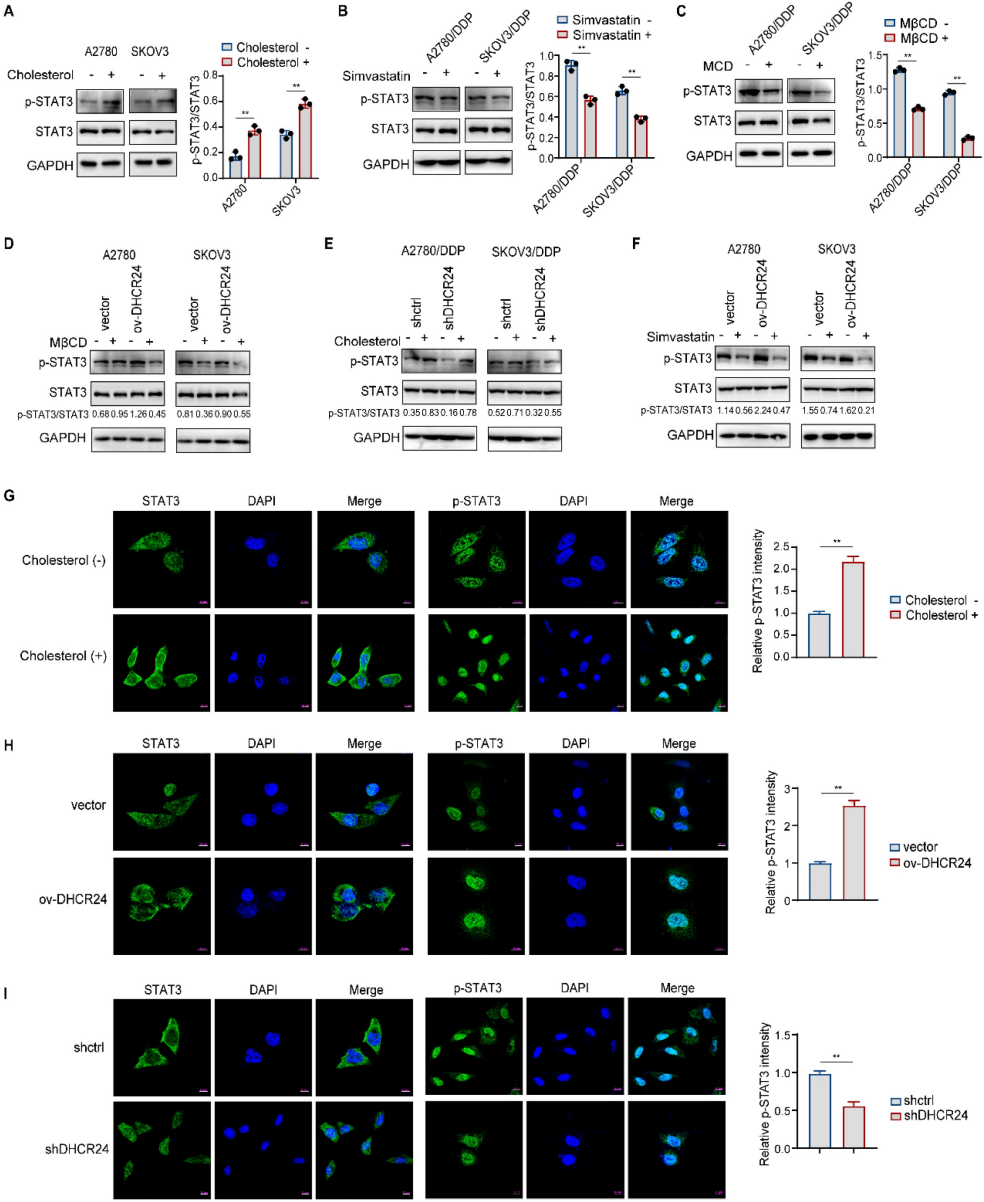
** DHCR24 regulates STAT3 activation through cholesterol synthesis. (A)** STAT3 phosphorylation in ovarian cancer cells treated with exogenous cholesterol. **(B)** STAT3 activation in drug-resistant cells following Simvastatin treatment. **(C)** Suppression of STAT3 phosphorylation upon lipid raft disruption with MβCD in drug-resistant cells. **(D)** MβCD abrogated STAT3 activation in DHCR24-overexpressing cells. **(E)** Cholesterol rescued STAT3 phosphorylation in DHCR24-silenced drug-resistant cells. **(F)** Simvastatin inhibited STAT3 activation in DHCR24-overexpressing cells. **(G)** Immunofluorescence analysis of STAT3 and p-STAT3 subcellular localization in cholesterol-treated A2780 cells. **(H)** STAT3 and p-STAT3 localization in A2780 DHCR24-overexpressing and control cells. **(I)** STAT3 and p-STAT3 distribution in DHCR24-knockdown A2780/DDP drug-resistant cells. All data are shown as the mean ± SD; ** p < 0.01, n = 3.

Our previous results demonstrated that DHCR24 upregulates cholesterol synthesis and promotes STAT3 signaling activation. Therefore, we consider whether the promotion of STAT3 signaling pathway activation by DHCR24 is related to cholesterol synthesis. Existing studies indicate that cholesterol promotes STAT3 activation in pancreatic cancer while its uptake inhibition suppresses STAT3 in myeloma, though mechanisms remain undefined. We observed that exogenous cholesterol increased STAT3 phosphorylation in ovarian cancer cells (Fig. 7A), and statin-mediated cholesterol synthesis inhibition suppressed STAT3 activation in drug-resistant cells (Fig. 7B), confirming cholesterol-dependent STAT3 regulation. Given the localization of upstream proteins of the STAT3 pathway within the raft, we used MβCD to disrupt lipid rafts, significantly inhibiting STAT3 phosphorylation (Fig. 7C). Notably, DHCR24 overexpression-induced STAT3 activation was abolished upon raft disruption (Fig. 7D), indicating DHCR24 requires raft integrity for STAT3 activation regulation. Mechanistically, cholesterol supplementation reversed STAT3 inhibition from DHCR24 silencing (Fig. 7E), and DHCR24 upregulation failed to activate STAT3 when *de novo* cholesterol synthesis was blocked (Fig. 7F). Additionally, immunofluorescence results demonstrated that cholesterol treatment (Fig. 7G) or DHCR24 overexpression (Fig. 7H) enriched STAT3 localization to membrane-proximal regions and enhanced nuclear accumulation of p-STAT3. Conversely, DHCR24 knockdown in drug-resistant cells (Fig. 7I) reduced membrane-proximal STAT3 localization and significantly diminished nuclear p-STAT3. Collectively, these findings, together with existing studies, suggest that DHCR24-driven cholesterol synthesis stabilizes cell membrane lipid raft microdomains, which may provide a critical signaling assembly platform for upstream activating kinases of STAT3. This inference is further supported by our observation that JAK2 is highly activated in chemoresistant cells (Fig. S6A) and that its phosphorylation is significantly suppressed upon DHCR24 knockdown (Fig. S6B).

### DHCR24-mediated activation of STAT3 signaling further upregulates DHCR24 expression

**Figure 8:**
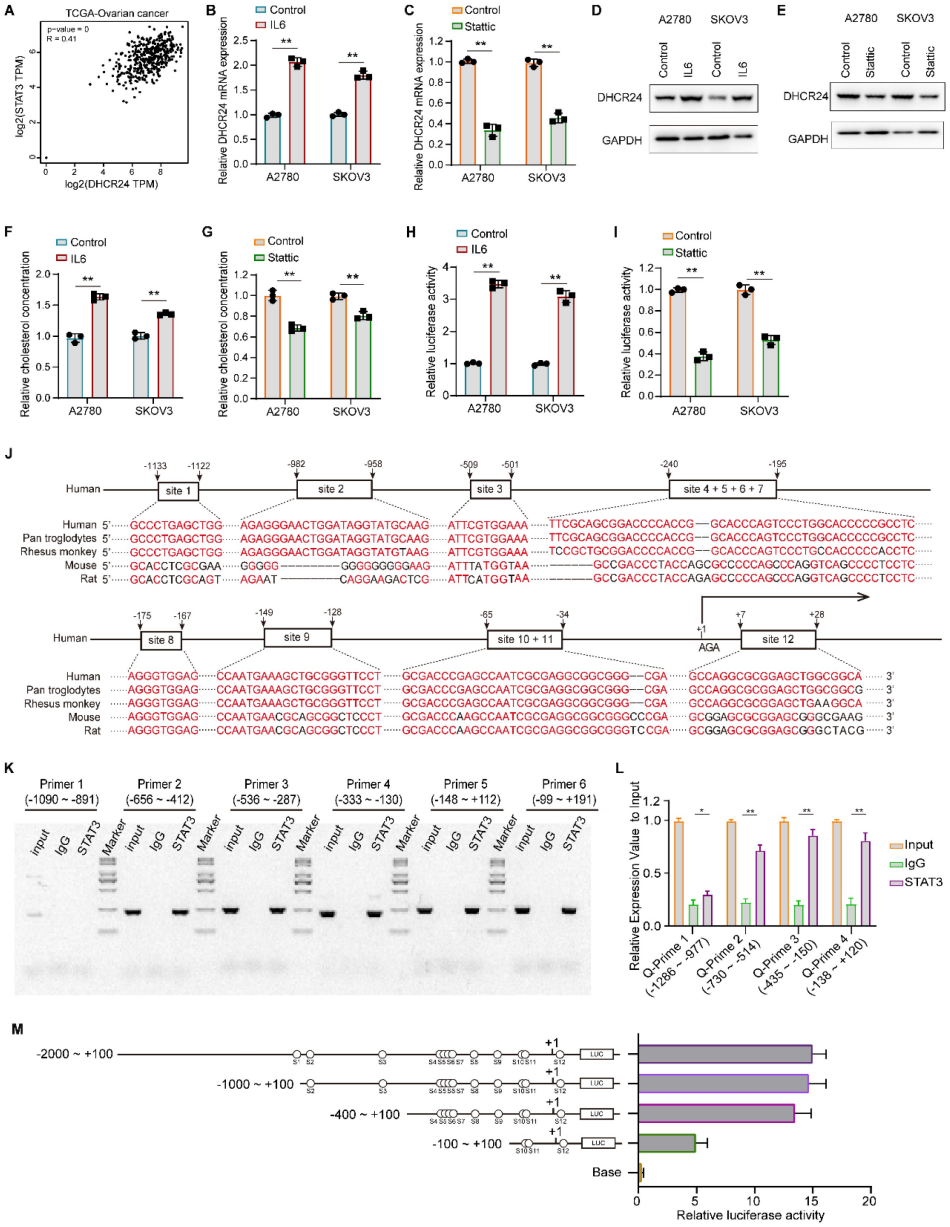
** STAT3 transcriptionally upregulates DHCR24 expression and modulates cellular cholesterol levels. (A)** Analysis of the TCGA database shows a significant positive correlation between STAT3 and DHCR24 mRNA expression in ovarian cancer. **(B and C)** Relative mRNA expression of DHCR24 in ovarian cancer cells following (B) IL-6-induced STAT3 activation or (C) STAT3 inhibition with Stattic. **(D and E)** DHCR24 protein levels in ovarian cancer cells after (D) IL-6 treatment or (E) Stattic treatment. **(F and G)** Cellular cholesterol concentration in ovarian cancer cells after (F) IL-6 treatment or (G) Stattic treatment. **(H and I)** Luciferase reporter assays demonstrating the effect of (H) IL-6 or (I) Stattic treatment on DHCR24 promoter activity. **(J)** Evolutionary conservation of the predicted STAT3 binding sites within the DHCR24 promoter across five species. **(K)** ChIP-PCR validation of STAT3 binding to the DHCR24 promoter region using specific primer sets. **(L)** ChIP-qPCR analysis demonstrating significant enrichment of STAT3 on the DHCR24 promoter region. **(M)** Schematic of the DHCR24 promoter-luciferase construct. All data are shown as the mean ± SD; * p < 0.05, ** p < 0.01, n = 3.

Analysis of the TCGA database revealed a significant positive correlation between STAT3 and DHCR24 at the mRNA level (Fig. 8A). We therefore investigated whether STAT3 activation could regulate DHCR24 gene expression in ovarian cancer. Our results demonstrated that IL-6-induced STAT3 activation promoted DHCR24 expression in ovarian cancer cells (Fig. 8B and D), whereas inhibition of STAT3 with Stattic significantly downregulated DHCR24 levels (Fig. 8C and E). These changes were accompanied by alterations in cellular cholesterol concentration, IL-6 increased cholesterol levels (Fig. 8F), while Stattic reduced them (Fig. 8G). These findings indicate that STAT3 activation indeed promotes DHCR24 expression. Furthermore, luciferase reporter assays confirmed that STAT3 activation positively regulates DHCR24 expression (Fig. 8H), whereas Stattic negatively regulates it (Fig. 8I). Furthermore, bioinformatic analysis using Animal TFDB v4.0 (https://guolab.wchscu.cn/AnimalTFDB4/#/) and the canonical STAT3 binding motif identified STAT3 as a putative transcription factor for the DHCR24 promoter, with 12 candidate binding sites on the forward strand (Fig. S4 and Supplementary Table 1). These sites showed evolutionary conservation across five species (Fig. 8J). Experimental validation by ChIP and PCR using primers (Fig. S7 and Supplementary Table 2) spanning the -656 to +191 region confirmed STAT3 binding, as evidenced by distinct amplification bands from multiple primer sets (Fig. 8K). Subsequent PCR analysis of the -730 to +200 region further demonstrated significant enrichment of STAT3 on the DHCR24 promoter (Fig. 8L). Furthermore, the results of dual-luciferase activity analysis confirmed the regulatory effect of STAT3 on DHCR24 (Fig. 8M). Collectively, these complementary computational and experimental approaches establish that STAT3 binds specifically and with high affinity to defined regions within the DHCR24 promoter.

## Discussion

This study identified DHCR24 as a central regulatory factor for chemotherapy resistance in ovarian cancer, functioning through a previously unrecognized self-reinforcing signaling pathway that combines cholesterol metabolism with the carcinogenic STAT3 signaling pathway. Our systematic research, from bioinformatics discoveries to functional validation of multiple experimental models, indicates that DHCR24 is not only a powerful biomarker of platinum resistance but also actively promotes multidrug resistance phenotypes through cholesterol-mediated mechanisms. The consistency of the results we observed in established cisplatin-resistant cell lines, patient-derived primary cells and clinical tumor specimens provides compelling evidence that DHCR24 plays a fundamental role in treatment failure.

We have clarified the causal relationship between DHCR24 expression and chemotherapy resistance in ovarian cancer through comprehensive functional acquisition and functional loss studies. The absence of DHCR24 significantly sensitized ovarian cancer cells to multiple chemotherapy drugs such as cisplatin, paclitaxel, and epirubicin, while its overexpression induced broad-spectrum drug resistance. This model indicates that the involvement of DHCR24 extends beyond platinum-specific resistance, including multidrug resistance mechanisms, positioning it as a major regulator of therapeutic response. Our findings have gained additional significance against the backdrop of recent parallel surveys 22-24, 28, 29. Although we have focused on the role of DHCR24 in chemotherapy resistance, studies have revealed that it promotes equally important functions such as proliferation, migration, invasion and epithelial-mesenchymal transition through the activation of the TGF-β1 pathway, jointly establishing the multifaceted oncoprotein status of DHCR24 in the pathogenesis of ovarian cancer 28.

Mechanistically, we found that DHCR24 mainly stably exerts its pro-drug resistance function through the lipid raft microdomain dependent on cholesterol synthesis. This discovery provides a key missing link between cholesterol metabolism and chemotherapy resistance, a link that remains poorly characterized in ovarian cancer. The significant increase in cholesterol levels in drug-resistant cells, coupled with rescue experiments indicating that exogenous cholesterol supplementation reversed the knockdown effect of DHCR24, while statins inhibited and mimicked them, jointly established that DHCR24-mediated cholesterol production is necessary and sufficient for endowing chemical resistance. Our research findings are consistent with the latest research results, which show that GDF15-induced chemotherapy resistance also increases the levels of ABCB1 and ABCC1 in the lipid membrane through DHCR24-mediated cholesterol metabolism 22. Therefore, our research further strongly verified that cholesterol metabolism is the core mechanism of DHCR24's function in the context of multiple drug resistance. It is worth noting that we chose to focus on P-gp for in-depth mechanism research, mainly due to its dependence on cholesterol and the lipid raft microenvironment. Existing literature indicates that the functions of MRP1/ABCC1 do not rely on cholesterol or cholesterol-stabilized lipid rafts, so their regulatory logic may differ from the mechanism revealed in this study. Therefore, this research aims to deeply elucidate a new lipid raft-dependent P-gp-specific regulatory pathway driven by the metabolic enzyme DHCR24, rather than comprehensively describing all drug resistance proteins.

Importantly, our research provides a key mechanism advancement, that is, clarifying how cholesterol-rich lipid rafts promote chemical resistance at the molecular level. We found that these special membrane domains serve as a platform for stabilizing P-gp through post-translational mechanisms. The specific damage to the stability of P-gp protein without corresponding mRNA changes, coupled with the weakened membrane localization caused by DHCR24 knockdown or cholesterol consumption, reveals a new regulatory mechanism that goes beyond the traditional focus on the transcriptional regulation of ABC transporters. This post-translational stabilization mechanism supplements the latest finding that the levels of ABCB1 and ABCC1 increase after the activation of the GDF15-DHCR24 axis, jointly providing a more comprehensive understanding of how metabolic reprogramming directly affects drug excretion capacity through quantitative and qualitative modifications to the excretion pump 22.

Beyond establishing the role of DHCR24 in drug efflux regulation, our work reveals its equally critical function in orchestrating STAT3 signaling activation through a mechanistically defined pathway. The essential requirement of intact lipid rafts for STAT3 activation, demonstrated through cholesterol depletion experiments using MβCD, positions DHCR24 as a key upstream modulator of this crucial survival pathway. Importantly, this finding aligns with and extends recent work demonstrating that spatial organization within sphingolipid/cholesterol-enriched membrane microdomains dictates Src-dependent STAT3 activation during epithelial cell competition and transformation 30-32. Our detailed analysis of STAT3 cellular trafficking provides further spatial context to this regulation, revealing pronounced STAT3 enrichment in membrane-proximal regions and enhanced nuclear translocation following either cholesterol supplementation or DHCR24 overexpression. This observed subcellular redistribution pattern supports a model in which DHCR24-derived cholesterol contributes to the formation and/or stabilization of lipid raft platforms, thereby creating a spatially organized microenvironment conducive to STAT3 phosphorylation and activation. Integrating our findings with established studies, we propose that DHCR24-driven cholesterol biosynthesis, by maintaining lipid raft integrity, establishes a critical membrane microdomain that likely facilitates the assembly and activity of upstream STAT3 kinases, including but not limited to JAK and Src family members.

Most significantly, our discovery that activated STAT3 transcriptionally upregulates DHCR24 expression completes a robust positive feedback loop that perpetuates and amplifies the chemoresistant phenotype. This DHCR24-STAT3 regulatory circuit, when integrated with the parallel discovery of DHCR24-mediated TGF-β1 pathway activation, firmly establishes DHCR24 as a nodal coordinator within ovarian cancer signaling networks. The convergence of these independent mechanistic studies demonstrates that DHCR24 occupies a privileged position at the intersection of metabolic reprogramming and oncogenic signaling, coordinating multiple pro-tumorigenic pathways through cholesterol-dependent organization of membrane signaling platforms. The identification of this sophisticated self-reinforcing circuit not only explains the persistence of chemoresistance but also highlights the particular vulnerability of this regulatory axis to therapeutic intervention.

The therapeutic significance derived from these comprehensive findings is substantial. It is worth noting that recent studies have shown that DHCR24 knockout is more effective than statin therapy in reversing chemotherapy resistance, suggesting that specific DHCR24 inhibition may outperform broad cholesterol synthesis blocking in terms of efficacy and potential toxicity reduction. This significant discovery, combined with our demonstration of the DHCR24-STAT3 self-reinforcing circuit, determines that DHCR24 is a particularly promising therapeutic target, and its inhibition can simultaneously disrupt multiple drug resistance mechanisms. The collection of evidence from independent research groups strongly supports the translational potential of targeting DHCR24 in the treatment of drug-resistant ovarian cancer. Although our research results have significantly promoted the understanding of the role of DHCR24 in chemical resistance, certain aspects are worthy of further study. For instance, the precise molecular components that promote STAT3 phosphorylation within the lipid raft microdomain remain to be fully characterized. Furthermore, the relationship between the STAT3 and TGF-β1 pathways in mediating multiple functions of DHCR24 offers an interesting field for future research, which can reveal additional regulatory layers. The clinical transability of targeting the DHCR24-axis, especially through the combination of specific inhibitors and conventional chemotherapy, is worthy of rigorous evaluation in advanced preclinical models.

**Figure 9:**
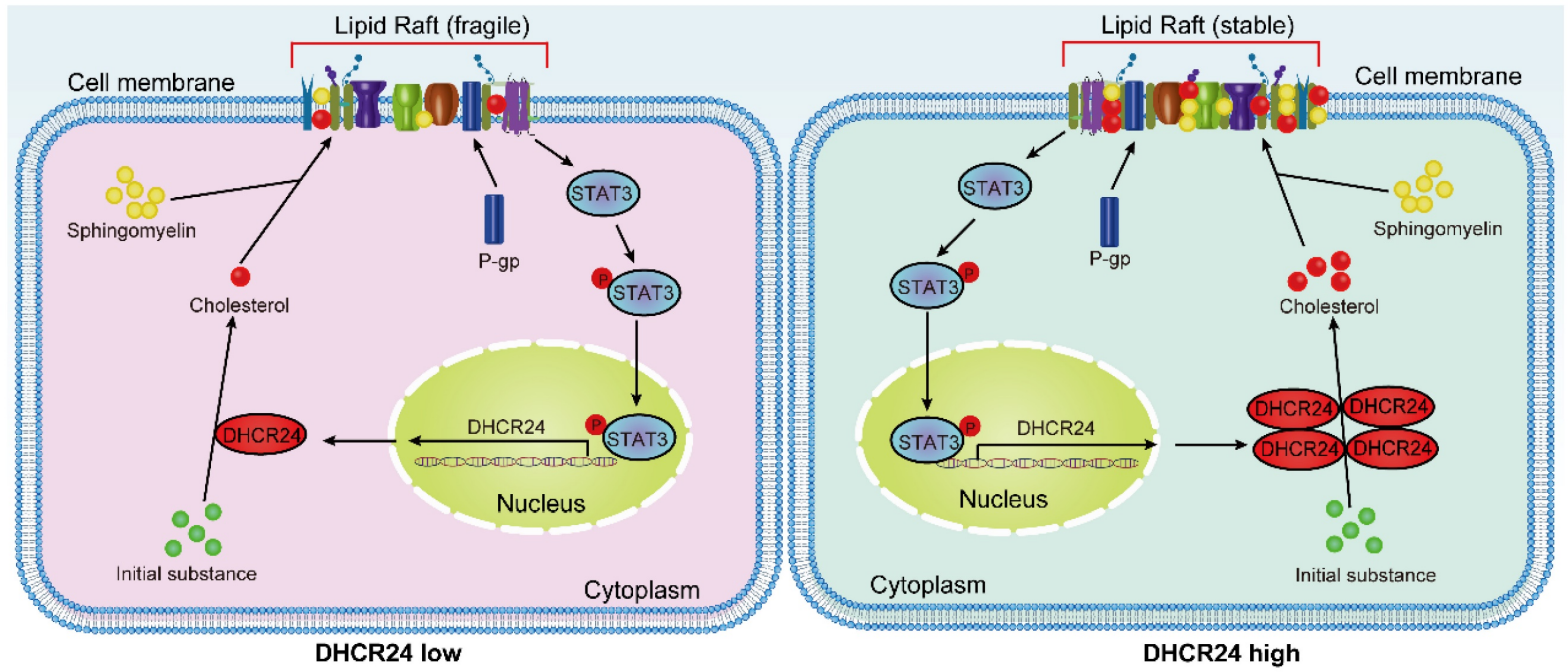
Proposed working model: DHCR24 enhances cholesterol biosynthesis, which stabilizes lipid raft microdomains to promote P-gp protein stability and facilitate STAT3 membrane recruitment and activation. Furthermore, activated STAT3 transcriptionally upregulates DHCR24 expression, establishing a positive feedback loop that perpetuates the chemoresistant phenotype.

In conclusion, our work, together with recent parallel findings, has elucidated the role of DHCR24 at the intersection of metabolic reprogramming and signal transduction in chemotherapy-resistant ovarian cancer. By demonstrating its dual effects of stabilizing P-gp and activating STAT3 through cholesterol-rich lipid rafts, while being transcriptionally regulated by STAT3, we have revealed a complex self-amplification circuit that maintains a resistant phenotype (Fig. 9). These shared insights not only promote our fundamental understanding of chemotherapy resistance in ovarian cancer, but also firmly establish DHCR24 as a high-value therapeutic target, whose inhibition can simultaneously disrupt multiple resistance mechanisms, offering new hope for overcoming the treatment failure of this devastating disease.

## Supplementary Material

Supplementary figures and tables.
