# Supplementary material for: DHCR24 Drives Ovarian Cancer Chemoresistance Through Lipid Raft-mediated P-gp Stabilization and STAT3 Activation
Source: Int J Biol Sci. 2026 Mar 17;22(7):3451–69. doi: 10.7150/ijbs.128173 (PMC13085885; doi:10.7150/ijbs.128173)
Supplement: Supplementary file 1 — Supplementary figures and tables. [file ijbsv22p3451s1.pdf]

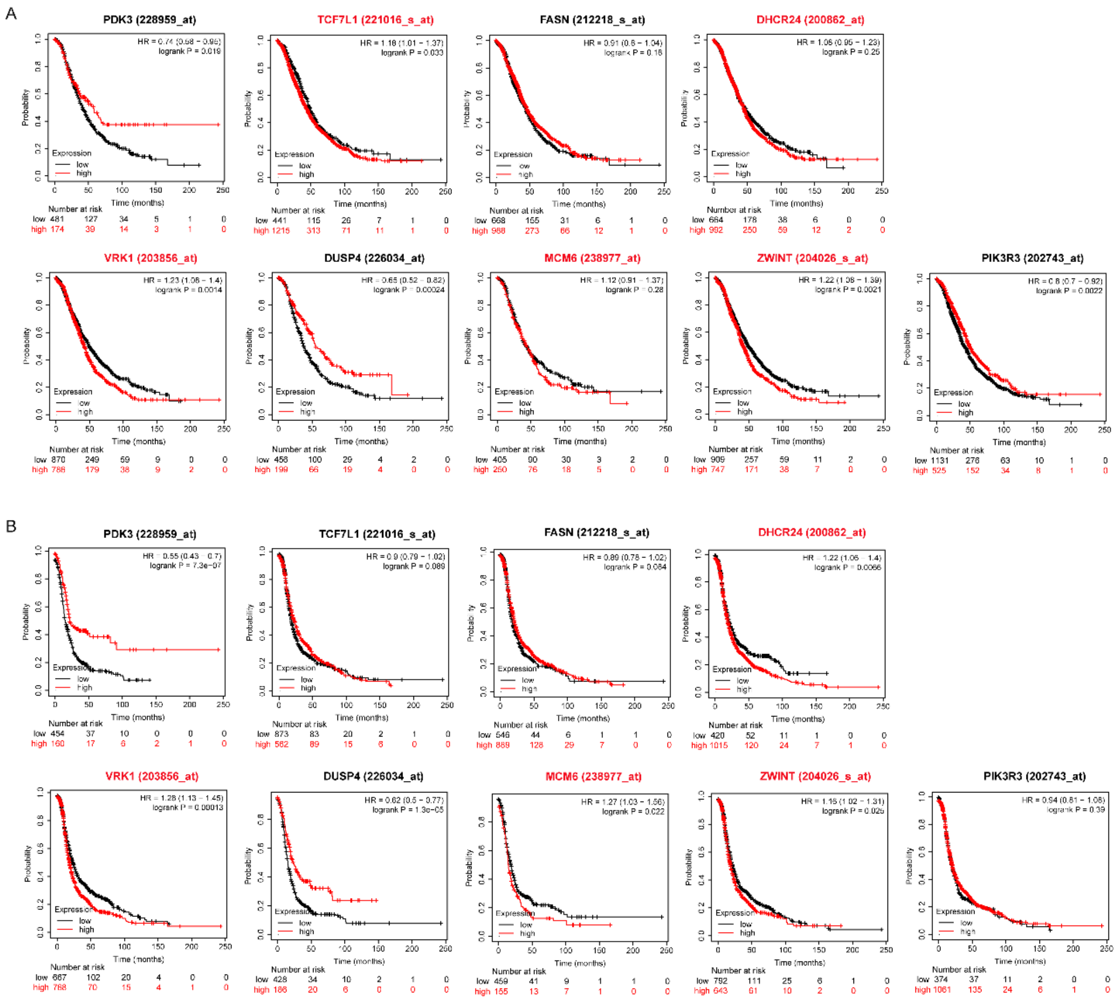

Fig. S1 Survival analysis of 9 candidate genes in ovarian cancer patients using the KMplot database. A, Overall survival (OS) based on high versus low expression of the 9 genes. B, Progression-free survival (PFS) based on high versus low expression of the 9 genes.

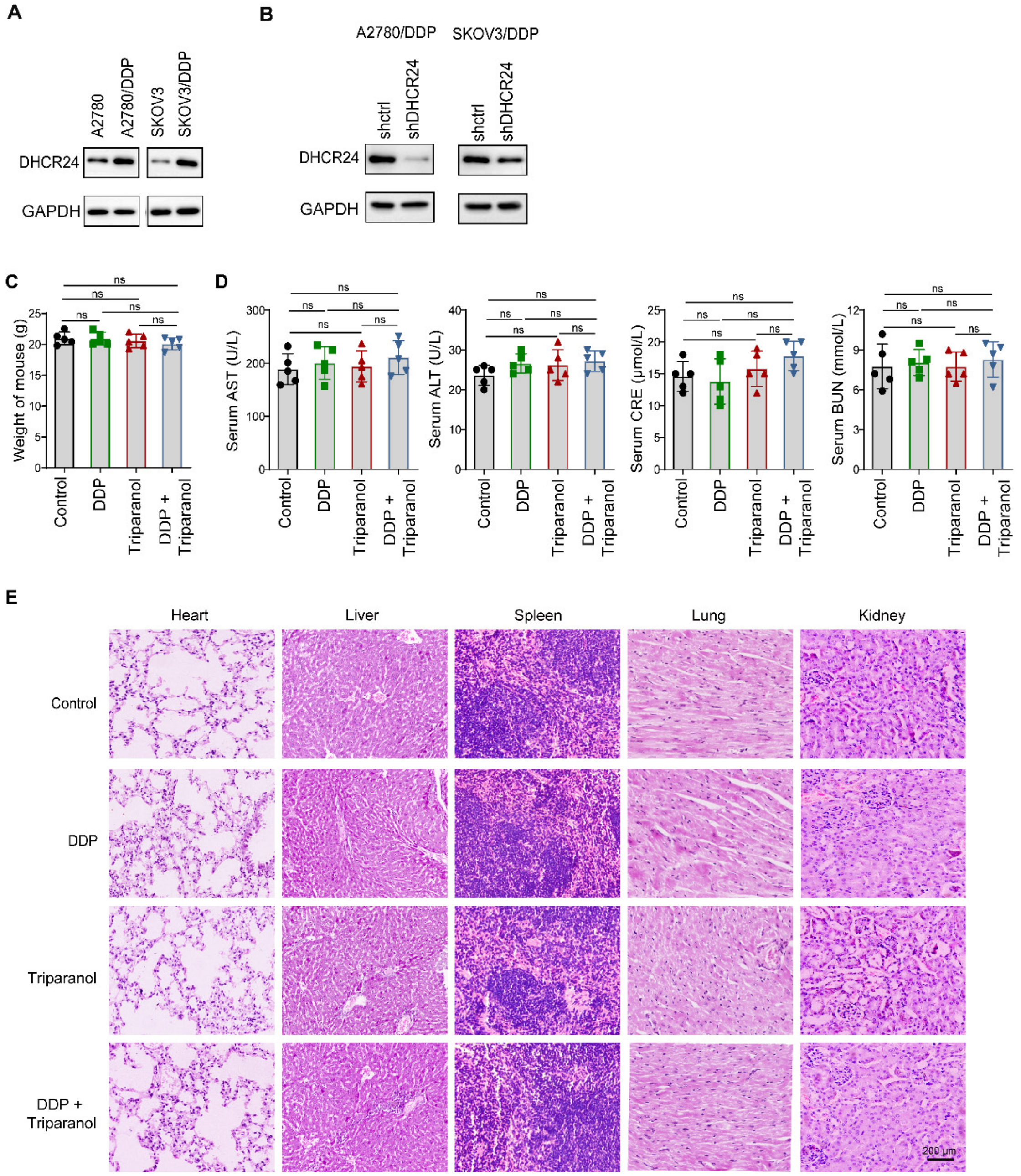

Fig. S2 The expression level of DHCR24 protein in drug-resistant cells and the potential toxicity of DHCR24 inhibition on normal mouse tissues. A, DHCR24 protein levels in drug-resistant ovarian cancer cells versus parental cells. B, Validation of DHCR24 knockdown efficiency in drug-resistant cells by Western blot. C, Body weights of mice during the treatment period. D, Serum biochemistry analysis. Levels of liver function markers (ALT, AST) and renal function markers (CRE, BUN) all remained within normal physiological ranges. E, Representative H&E-stained sections of vital organs (heart, liver, spleen, lung, and kidney). All data are shown as the mean ± SD; ns, non-significant.

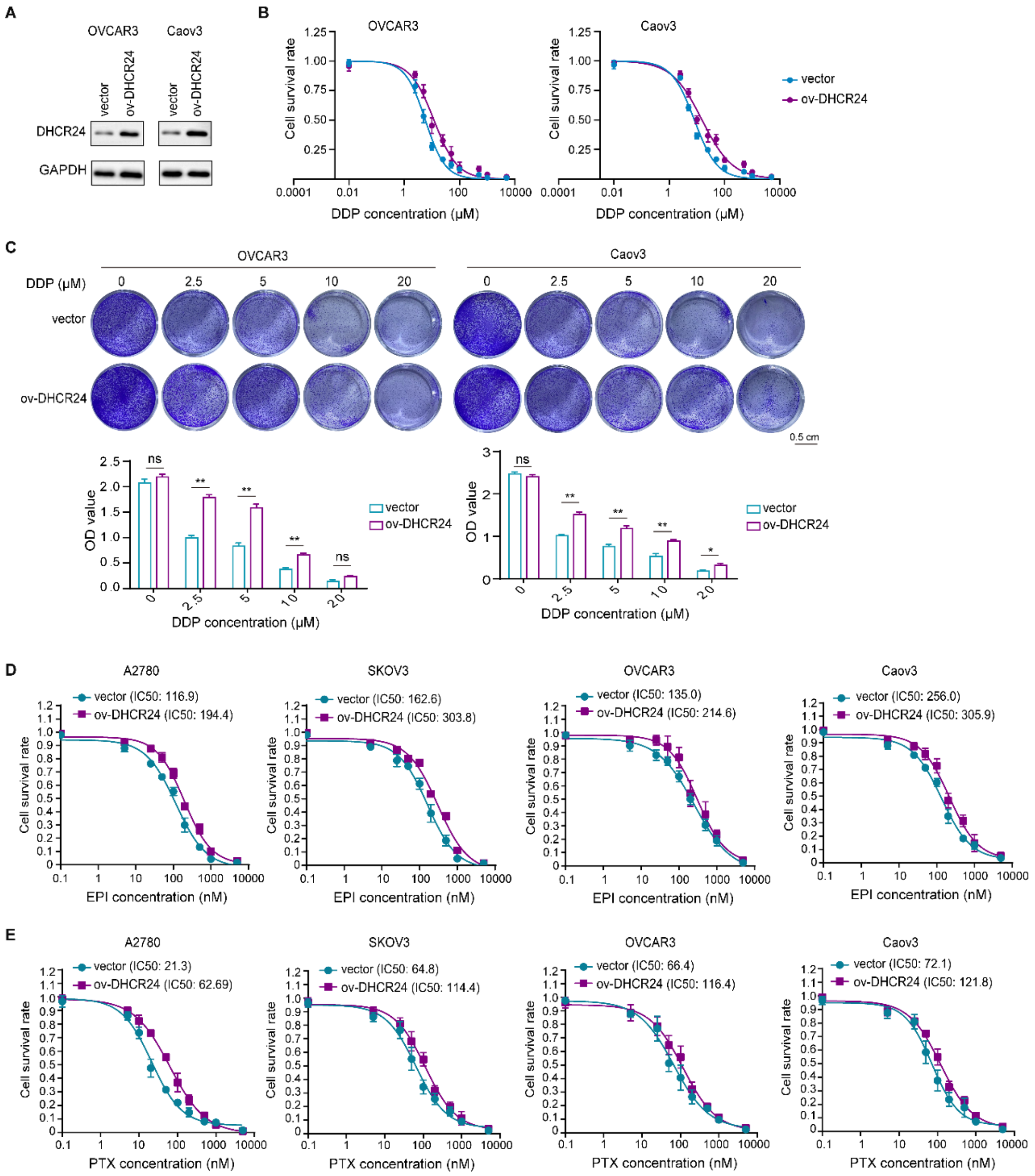

Fig. S3 Modulation of DHCR24 expression affects chemosensitivity in ovarian cancer cells. A, Validation of DHCR24 overexpression in ovarian cancer cells by Western blot. B, IC50 values of DDP at 48 h in DHCR24-overexpressing cells. C, Effect of DHCR24 overexpression on DDP sensitivity detected by cell proliferation assay. D, IC50 values of epirubicin (EPI) at 48 h in DHCR24-overexpressing and control cells. E, IC50 values of paclitaxel (PTX) at 48 h in DHCR24-overexpressing and control cells. All data are shown as the mean ± SD; ns, non-significant, * $p < 0.05$, ** $p < 0.01$, $n = 3$.

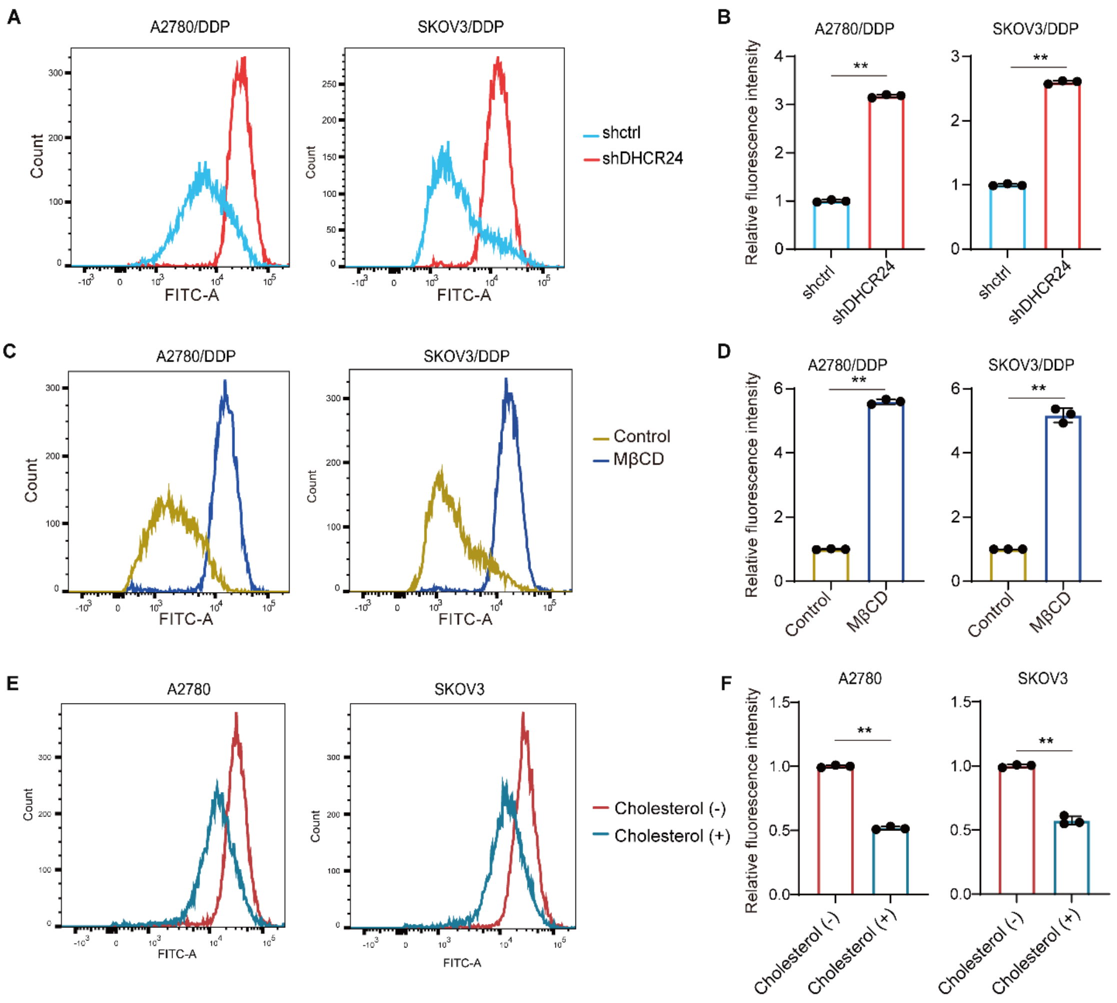

Fig. S4. DHCR24 knockdown or membrane cholesterol depletion enhances intracellular Rhodamine 123 accumulation. A, Flow cytometry analysis of Rhodamine 123 accumulation in cells following DHCR24 knockdown. B, Quantification of the mean fluorescence intensity shown in (A). C, Flow cytometry analysis of Rhodamine 123 accumulation in cells treated with MβCD. D, Quantification of the mean fluorescence intensity shown in (C). E, Flow cytometry analysis of Rhodamine 123 accumulation in cells treated with cholesterol. F, Quantification of the mean fluorescence intensity shown in (E). All data are shown as the mean ± SD; ** $p < 0.01$, $n = 3$.

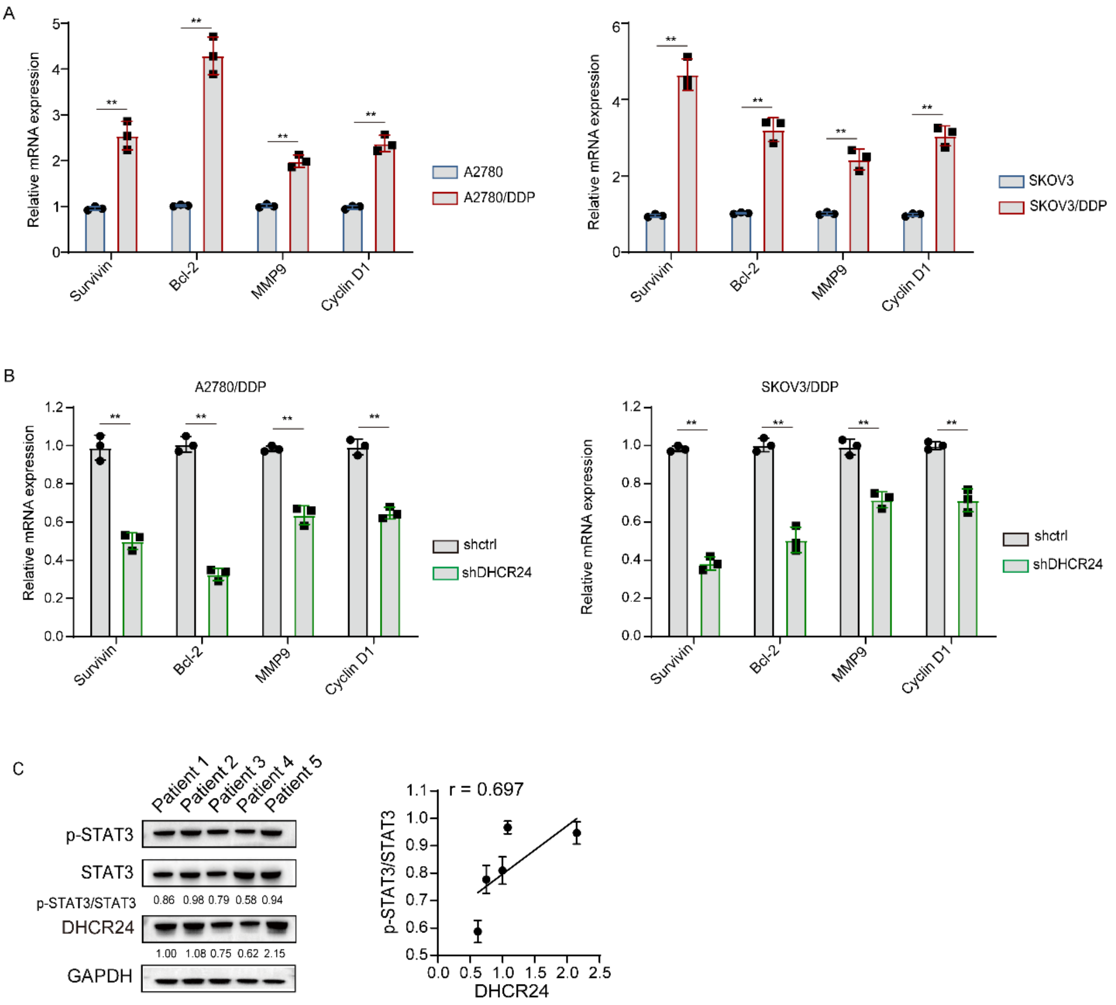

Fig. S5 DHCR24 modulates chemosensitivity through STAT3 signaling in ovarian cancer. A, The expression levels of STAT3-regulated genes (Survivin, MMP9, cyclinD1, BCL-2) in drug-resistant cells and parental cells. B, The effect of silencing DHCR24 on the expression level of Survivin, MMP9, cyclinD1, BCL-2 genes in drug-resistant ovarian cancer cells. C, A significant positive correlation between DHCR24 and p-STAT3 protein levels was confirmed in primary ovarian cancer cells. All data are shown as the mean ± SD; ** $p < 0.01$, $n = 3$.

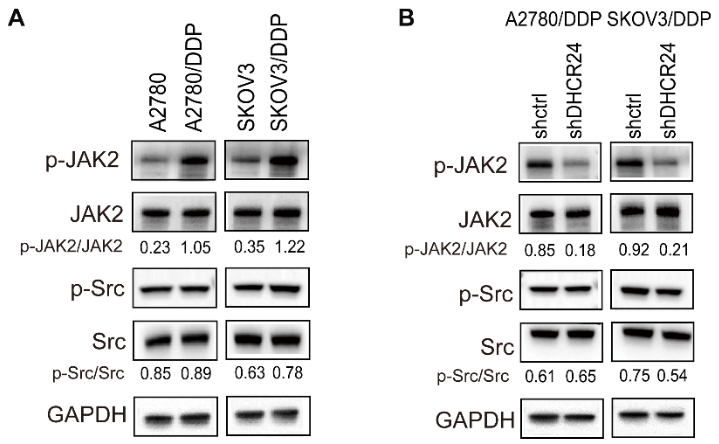

Fig. S6 DHCR24 regulates JAK2 activation in chemoresistant ovarian cancer cells. A, p-JAK2 is markedly elevated in chemoresistant cells compared to their parental counterparts. B, Knockdown of DHCR24 significantly suppresses JAK2 phosphorylation in chemoresistant cells.

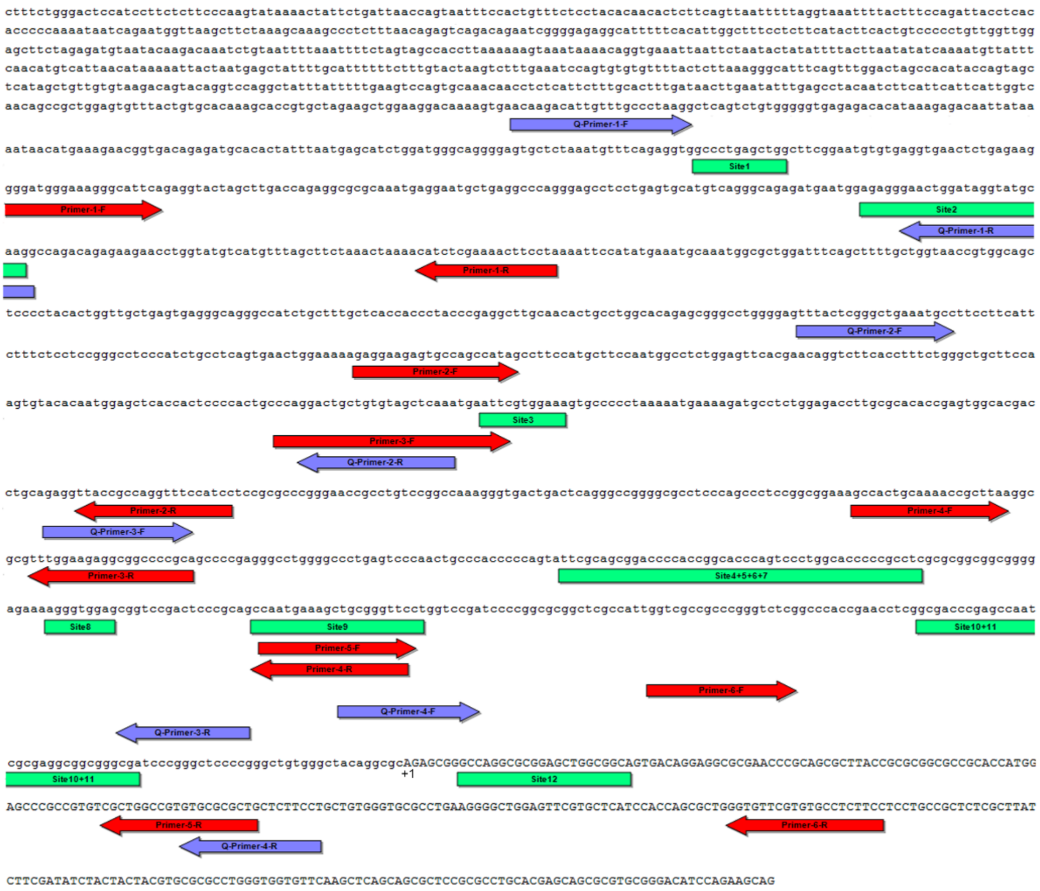

Fig. S7 12 candidate binding sites on the forward strand.

Supplementary Table 1 Sequence of primers used for qRT-PCR

Name	Primer	Sequence
DHCR24	Upper	5'-GGCAACAACCCCATCTTCC-3'
	Lower	5'-GACCTGGCTTCAAAGTGTTTC-3'
VRK1	Upper	5'-ACGATGGCACTATTGAATTCACGAGC-3’
	Lower	5'-AATTATCCTCCCAAGGAAGATGGC-3’
MCM6	Upper	5'-GAGGACTTCATTGTGGAGCA-3'
	Lower	5'-TTCAGTAACCGGAAAGCTTCC-3’
ZWINT	Upper	5'-GAGAAGCATCTGCAGCATCT-3'
	Lower	5'-AGGTTTCCAAGTTTCTGAAACACC-3’
Survivin	Upper	5'-GACGACCCCATAGAGGAAC-3’
	Lower	5'-TGCAATTTTGTTCTTGGCTCTTTCTC-3’
MMP9	Upper	5'-TTTGAGGAGCGGCTCTCCAAG-3’
	Lower	5'-TCTTCCCCCTGCCACTCC-3’
Cyclin D1	Upper	5'-CTAAGATGAAGGAGACCATCC-3’
	Lower	5'-AAGTGTTCAATGAAATCGTGCGG-3’
BCL-2	Upper	5'-GCGTCAACCGGGAGATGT-3’
	Lower	5'-CAAACTGAGCAGAGTCTTCAG-3’
β-actin	Upper	5'-GAAATCGTGCGTGACATTAA-3’
	Lower	5'-AAGGAAGGCTGGAAGAGTG-3’

Supplementary Table 2 Bioinformatic analysis using Animal TFDB v4.0 and the canonical STAT3 binding motif

Site	start	stop	Score	p value	q value	Matched Sequence
1	-1133	-1122	10.7143	8.64E-05	0.366	GCCCTGAGCTGG
2	-982	-958	15.2532	1.95E-06	0.0089	AGAGGGAACTGGATAGGTATGCAAG
3	-509	-501	-	-	-	ATTCGTGGAAA
4	-240	-219	11.5429	2.94E-05	0.0799	TTCGCAGCGGACCCCACCGGCA
5	-233	-212	9.24286	8.82E-05	0.0799	CGGACCCCACCGGCACCCAGTC
6	-223	-202	9.01429	9.76E-05	0.0799	CGGCACCCAGTCCCTGGCACCC
7	-203	-195	13.9143	1.07E-05	0.0456	CCCCGCCTC
8	-175	-167	11.6974	6.59E-05	0.137	AGGGTGGAG
9	-149	-128	11.1818	5.06E-05	0.0681	CCAATGAAAGCTGCGGGTTCCT
10	-65	-44	7.96053	9.49E-05	0.089	GCGACCCGAGCCAATCGCGAGG
11	-55	-34	14.2078	6.57E-06	0.0265	CCAATCGCGAGGCGGCGGGCGA
12	7	28	11.6053	1.77E-05	0.0221	GCCAGGCGCGGAGCTGGCGGCA

Supplementary Table 3 The primers used in ChIP Assay

Name	Sequence
Primer-1-F	5'-GGGATGGGAAAGGGCATTCA-3'
Primer-1-R	5'-TAGGAAGTTTTCGAGATG-3'
Primer-2-F	5'-GAGGAAGAGTGCCAGCCATAG-3'
Primer-2-R	5'-GGATGGAAACCTGGCGGTAA-3'
Primer-3-F	5'-CCAGGACTGCTGTGTAGCTCAAATGAATTC-3'
Primer-3-R	5'-TGCGGGGCCGCCTCTTCCAAA-3'
Primer-4-F	5'-GCCACTGCAAAACCGCTTAA-3'
Primer-4-R	5'-GAACCCGCAGCTTTCATTGG-3'
Primer-5-F	5'-CAATGAAAGCTGCGGGTTCC-3'
Primer-5-R	5'-CAGCGCGCACACGGCCAGCG-3'
Primer-6-F	5'-GGTCGCCGCCCGGGTCTCG-3'
Primer-6-R	5'-GGAAGAGGCACACGAACACC-3'
Q-Primer-1-F	5'-ACAAGACATTGTTTGCCCTAAGG-3'
Q-Primer-1-R	5'-CCTTGCATACCTATCCAGTTC-3'

Q-Primer-2-F	5'-TTTACTCGGGCTGAAATGCC-3'
Q-Primer-2-R	5'-TTTGAGCTACACAGCAGTCC-3'
Q-Primer-3-F	5'-GAGGTTACCGCCAGGTTTC-3'
Q-Primer-3-R	5'-CGGTCCGACTCCCGCAG-3'
Q-Primer-4-F	5'-TGCGGGTTCCTGGTCCGA-3'
Q-Primer-4-R	5'-AGGAAGAGCAGCGCGCAC-3'
